# Preclinical activity of brincidofovir in peripheral T-cell and NK/T-cell lymphoma

**DOI:** 10.1186/s12916-026-04680-8

**Published:** 2026-02-06

**Authors:** Jason Yongsheng Chan, Elizabeth Chun Yong Lee, Kelila Xin Ye Chai, Boon Yee Lim, Zhimei Li, Jing Yi Lee, Bavani Kannan, Hui Yi Tay, Tun Kiat Ko, Jessica Sook-Ting Kok, Kah Suan Lim, Nur Ayuni Binte Muhammad Taib, Dachuan Huang, Jing Quan Lim, Masatoshi Hazama, Koji Fukushima, Bin Tean Teh, Soon Thye Lim, Choon Kiat Ong

**Affiliations:** 1 Cancer Discovery Hub, National Cancer Centre Singapore, Singapore, Singapore; 2 Division of Medical Oncology, National Cancer Centre Singapore, Singapore, Singapore; 3 Duke-NUS Medical School, Singapore, Singapore; 4 Lymphoma Translational Research Laboratory, Division of Cellular and Molecular Research, National Cancer Centre Singapore, Singapore, Singapore; 5 Laboratory of Cancer Epigenome, National Cancer Centre Singapore, Singapore, Singapore; 6 SymBio Pharmaceuticals Limited, Tokyo, Japan

**Keywords:** NK/T-cell lymphoma, Nucleoside analogue, Replication stress, Immunotherapy, PD-L1

## Abstract

**Background:**

Brincidofovir (BCV) is a novel nucleoside phosphonate analogue with unique dual antiviral and anti-tumor properties.

**Methods:**

The activity of BCV was evaluated in 44 cell-line models, including T/NK-cell non-Hodgkin lymphoma (T/NK-NHL, *n* = 25) and B-cell lymphoma (BCL, *n* = 19), and their respective NOD/SCID mice xenograft models. The potential immunogenic effects were examined in a syngeneic EL4-C57BL/6 murine lymphoma model.

**Results:**

BCV demonstrated potent anti-tumor activity across the majority of cell lines regardless of EBV positivity, with IC50 values within clinically achievable human plasma concentrations (2 µg/ml) in 17 of 25 (68.0%) T/NK-NHL and in 13 of 19 (68.4%) BCL. In vivo treatment significantly inhibited tumor growth in all xenograft models compared to vehicle control. Notably, RNAseq analysis demonstrated BCV downregulated MYC-target pathways in T/NK-NHL models. BCV evoked S-phase cell cycle arrest, replication stress, DNA damage, and apoptosis while triggering STING pathway-mediated interferon responses, PD-L1 expression, and immunogenic cell death. In the EL4-C57BL/6 model, BCV in combination with anti-PD1 significantly inhibited tumor growth and triggered an immune reaction characterized by the highest scores for adaptive immune response, cytokines/chemokines and receptors, cytotoxic cells, dendritic cells, NK CD56dim cells, and neutrophils (NanoString Immunology Panel).

**Conclusions:**

Taken together, these results demonstrate a novel role for BCV in lymphoma therapy and suggest potential for combination with checkpoint immunotherapy.

**Supplementary Information:**

The online version contains supplementary material available at 10.1186/s12916-026-04680-8.

## Background

The peripheral T-cell lymphomas (PTCL) and natural killer/T-cell lymphomas (NKTCL) represent groups of rare, aggressive non-Hodgkin lymphomas with poor prognoses. Collectively, they are more prevalent in certain ethnogeographic regions, including East Asia, and represent a major healthcare need. Overall survival outcomes have not significantly improved, save for the introduction of brentuximab vedotin for the subset of CD30-positive PTCL in the frontline setting and L-asparaginase-based regimens for NKTCL, as compared with historically adopted anthracycline-based “CHOP”-like regimens [1]. In the salvage setting for relapsed/refractory disease, novel agents such as epigenetic therapies have been approved for PTCL [2], albeit with limited efficacy, while no standard effective treatment currently exists for NKTCL beyond conventional chemotherapy [3].

Previously, certain nucleoside analogues have been suggested to elicit viral-independent anti-tumor activity, in addition to their known antiviral effects. In particular, cidofovir (CDV), an acyclic nucleoside phosphonate with a broad target range of viral species including the Epstein-Barr virus (EBV), was initially shown to be effective against EBV-related malignancies such as Burkitt lymphoma and nasopharyngeal carcinoma in cell-line and xenograft models [4, 5]. This anti-tumor effect was mediated through apoptosis and was accompanied by downregulation of EBV-related oncoproteins LMP1 and EBNA2A [5]. Subsequent studies have further demonstrated promising efficacy and safety of topical CDV injections in two patients with locally recurrent nasopharyngeal carcinoma [6], as well as intracavitatory administration of CDV in herpesvirus-associated primary effusion lymphoma [7]. More recently, CDV was further demonstrated to evoke anticancer activity in viral-negative tumors through the induction of DNA damage as a mechanism of action [8–10], which led us to a working hypothesis that CDV may represent a unique agent with dual antiviral and anti-tumor properties while also being potentially immune-activating as a consequence of DNA damage [11].However, as its toxicity profile for systemic administration at doses achieving therapeutically-tractable drug concentrations would preclude feasibility, an alternative strategy would be required for clinical translation.

Brincidofovir (BCV) is a novel lipid conjugate of CDV with improved intracellular delivery, higher potency, and a favorable toxicity profile compared to CDV and carries significantly reduced risks of nephrotoxicity and myelosuppression. BCV is known to convert to CDV intracellularly after cleavage of its lipid moiety, which then undergoes di-phosphorylation into its active form. While the orally active formulation is currently approved as a countermeasure against smallpox, the intravenous formulation has been undergoing development for the treatment of adenovirus infection in allogeneic stem cell transplant recipients, given its more favorable gastrointestinal toxicity profile [12, 13]. Unlike CDV, however, the role of BCV in cancer treatment has not been evaluated. Therefore, in this study, we comprehensively investigated the efficacy of BCV in a panel of non-Hodgkin lymphoma cell-line and xenograft models, as well as the mechanisms underlying its anti-tumor activity, paving the way for an early phase clinical trial in patients with lymphoma.

## Methods

### Patient data and biospecimen collection

The clinical information and archival formalin-fixed paraffin-embedded (FFPE) tissue samples of patients diagnosed with NKTCL were obtained from the National Cancer Centre Singapore (Additional file 2: Table S3). All cases were reviewed by expert hematopathologists at the Singapore General Hospital. Tissue collection and consent protocols were under ethics approval by the SingHealth Centralized Institutional Review Board (CIRB 2018/3084). Written informed consent from the patients for the use of clinical data and biospecimens was obtained in accordance with the Declaration of Helsinki.

### Cell lines, reagents, and quantification of viability

Forty-five cell lines were included in our study, including NKTCL (*n* = 11), B-cell lymphoma (*n* = 19), T-cell lymphoma (n = 14), and chronic myeloid leukemia (*n* = 1). The source and cell culture conditions are summarized in Additional file 2: Table S13. All cell lines were recently authenticated and regularly tested for mycoplasma contamination. BCV was obtained from AdooQ (no. A13326, Irvine, CA, USA), and prepared according to the manufacturer’s recommendations (dissolved in DMSO for in vitro experiments). Aciclovir, ganciclovir, adefovir, foscarnet, penciclovir, and CDV were obtained from Selleck Chemicals (Houston, TX, USA). Cell viability was quantified via Promega CellTiter-Glo® 2.0 Cell Viability Assay (Promega, Madison, WI, USA), as per the manufacturer’s protocol. Briefly, cells were seeded in 96-well plates at a concentration of 2 × 10^3^ cells in 100 μl of media per well. After 96 h, the Promega CellTiter-Glo® 2.0 Cell Viability Assay reagent was added to each well and incubated for 10 min at room temperature before measuring absorbance at 480 nm using the Tecan M200 Infinite 96-well plate reader with iControl software 1.6 (Tecan, Männedorf, Switzerland). Cell viability was assessed as the percentage of the mock-treated control absorbance. The growth inhibitory effects were analyzed by generating dose–response curves as a plot of the percentage of surviving cells versus drug concentration, and their IC50s were estimated using GraphPad Prism version 8.0.2 (GraphPad Software, Boston, MA, USA). All reactions were performed in triplicate.

### *X*enograft experiments and in vivo drug treatment

For in vivo drug treatment, lymphoma cell lines (NK-S1, PTCL-S1, PTCL-S2, and OCI-LY18) were subcutaneously implanted (0.5 × 10^6^ cells) into the flanks of 6-week-old female NSG mice. Similarly, murine EL4 cell lines were implanted into the flanks of 6-week-old female C57BL6N mice. Clinical-grade BCV (SymBio Pharmaceuticals, Tokyo, Japan) was intraperitoneally administered at a dosage of 40 mg/kg or vehicle control (5% dextrose in water) twice per week, starting after the tumors reached approximately 100 mm^3^ in size. The in vitro potency of BCV from SymBio was verified in the respective cell lines prior to in vivo experiments. The respective treatment subgroups received anti-PD-1 (200 µg twice per week) or isotype vehicle controls (no. BE0146 and no. BE0089, InVivoMAb, Lebanon, NH, USA). All animal studies were conducted in compliance with protocols approved by the SingHealth Institutional Animal Care and Use Committee (IACUC). A minimum of 8–10 mice were included per study group (treatment or control) for pragmatic reasons, unless otherwise stated (e.g., for the extended pilot study in B-cell lymphoma). After tumor engraftment, mice were assigned to the experimental groups after stratification by baseline tumor volume, followed by simple randomization. No blinding was done due to logistical and manpower constraints. Tumor measurements were recorded repeatedly until the vehicle control tumor sizes reached approximately 2000 mm^3^, when the mice were euthanized following institutional IACUC guidelines. Survival data was not available due to this constraint. Tumor sizes in experimental and control groups were averaged at each time point and compared statistically for all the mice.

### RNA isolation, whole transcriptome sequencing, and gene set enrichment analysis

Total RNA was extracted from tumor tissues and cell lines using the AllPrep DNA/RNA FFPE Kit and RNeasy Mini Kit, according to the manufacturer’s protocol (Qiagen, Valencia, CA, USA). The integrity of RNA was determined by electrophoresis using the 2100 Bioanalyzer and/or the 4200 TapeStation (Agilent Technologies, CA, USA). Whole transcriptome sequencing of cell lines was performed on Illumina platforms (NovogeneAIT Genomics, Singapore) using the standard Illumina RNA-seq protocol or on the MGI DNBSEQ-G400 platform using its standard MGI RNA-seq protocol (MGI Tech, China). Transcriptomic profiling of FFPE tumor tissue was performed using the Magnis SureSelect XT HS2 RNA Reagent Kit on the Magnis NGS Prep System (Agilent Technologies, CA, USA), followed by sequencing on the Illumina platform (NovogeneAIT Genomics, Singapore). Read alignment, transcript abundance estimation, identification of differentially expressed genes, and gene set enrichment analysis (GSEA) were performed as previously described [14]. A gene set is significantly enriched if its false discovery rate (FDR) *q*-value is below 0.05.

### Single-cell RNA sequencing

Single-cell RNA-seq (scRNAseq) libraries were prepared from NK-S1 and KAI-3 cell lines with (1 µg/ml for 48 h, 1 µg/ml for 72 h) or without treatment with BCV. Each cell was captured and uniquely barcoded using the 10 × Chromium Next GEM Single Cell 3′ Kit v3 (PN1000268) and 10 × Chromium Controller according to the manufacturer’s protocol (10 × Genomics, CA, USA). Briefly, an estimated 16,000 cells were loaded at a concentration of 1200 cells/μl in an attempt to recover 10,000 cells. Following Gel Beads-in-emulsion (GEMs) generation, cell lysis and dissolution of the Gel Bead within each reaction vesicle enabled reverse transcription of polyadenylated mRNA, producing cDNA tagged with both a universal cell barcode and a unique molecular index (UMI). The generated cDNA from each sample was used for 3′ gene expression (3′GEX) scRNA-seq library generation. Enzymatic fragmentation of the cDNA transcripts was carried out, followed by end repair and A-tailing, adaptor ligation, and final library amplification PCR with unique sample indices for each sample. Final library quality was determined using the Agilent Bioanalyzer High Sensitivity DNA Kit, and the libraries were sequenced on the Illumina platform (NovogeneAIT, Singapore).

### Analysis of single-cell sequencing data

The reads were demultiplexed and aligned against the GRCh38 reference genome using 10 × Cell Ranger v9.0.0 (10 × Genomics, CA, USA). The single-cell data were loaded into count matrices, and samples were merged based on their cell line identity, KAI-3 and NK-S1, using Seurat v5.2.1 [15]. A total of 17,051 and 9196 cells were retained for KAI-3 and NK-S1, respectively, after filtering for cells with less than 20% mitochondrial genes and expressed at least 450 genes. These cells were then subjected to scaling, log-normalization of gene expression measurements, principal component analysis with top 2000 genes selected by variance stabilizing transformation approach, construction of the nearest-neighbor graph using the first 15 principal components, and clustering of cells at 0.2 resolution with the Louvain algorithm as implemented by Seurat. UMAP was used to visualize the distribution of cells in the projection of the significant principal components. Differentially expressed genes were identified using a reference cluster and subsequently ranked based on the signed fold change and p-adjusted values. Significantly enriched pathways were determined using Fast Gene Set Enrichment Analysis, v1.30.0.

### Statistical analysis

Statistical analysis of mean values was performed using *t*-tests and one-way ANOVA, and results are presented as mean values and standard deviations. Median values were presented in Box-Whiskey plots and compared using Mann–Whitney *U*-tests. For survival analysis, high and low *TLE1* gene expression scores were dichotomized using receiver operative characteristic (ROC) curve analysis to derive the optimal cut-off values as a univariable predictor of overall survival (OS). Progression-free survival (PFS) was defined as the time from diagnosis until progression or death from any cause. OS was measured from diagnosis until the date of death from any cause or censored at last follow-up for survivors. Survival analyses were conducted using the Kaplan–Meier method and log-rank tests. All statistical tests assumed a two-sided test with a significance level of 0.05 unless otherwise stated and were performed using MedCalc for Windows version 19.0.7 (MedCalc Software, Ostend, Belgium).

## Results

### In vitro and in vivo activity of BCV in NKTCL

**Fig. 1:**
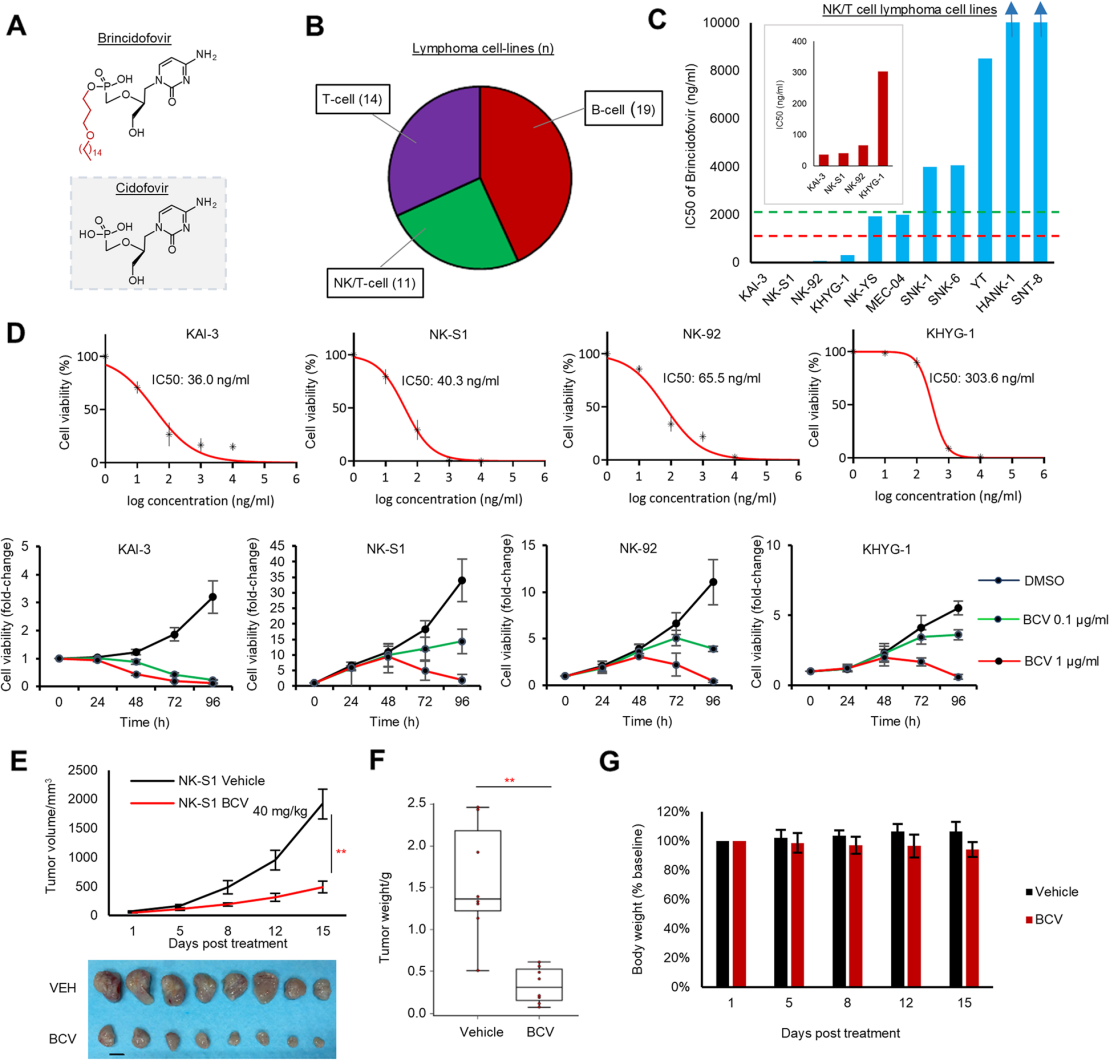
Repurposing brincidofovir for treatment of NK/T cell lymphoma. **A** Chemical structures of antiviral drugs cidofovir and brincidofovir (BCV). **B** Overview of lymphoma cell-line models used in the study. **C** IC50 of BCV across NKTCL cell lines including the EBV-negative KHYG-1 cell line. Inset: IC50 of four most sensitive cell lines. **D** BCV inhibits cell growth in NKTCL cell lines in a dose-dependent and time-dependent manner. Drug treatments were performed in triplicate, and results are presented as mean values and standard deviations. **E**, **F** BCV inhibited tumor growth in the NK-S1 xenograft model (tumor volume: *p* = 0.0005, two-tailed *t*-test; tumor weight: *p* = 0.0006, Mann–Whitney *U*-test). NSG mice were treated twice per week, IP, with either vehicle or BCV (40 mg/kg) 7 days after subcutaneous flank inoculation with 0.5 × 10^6^ NK-S1 cells (*n* = 8 per group) (scale bar: 10 mm). **G** No significant effect of BCV on mouse body weight was demonstrated

BCV, a novel lipid-conjugated nucleoside phosphonate analogue of CDV (Fig. 1A), was evaluated for its preclinical anti-lymphoma activity in a panel of 44 lymphoma cell-line models (NKTCL, *n* = 11; T-cell lymphoma, *n* = 14; B-cell lymphoma, *n* = 19) (Fig. 1B). BCV inhibited viability in all NKTCL cell lines in a dose- and time-dependent manner, with IC50 values within clinically achievable human plasma concentrations (2 µg/ml). Highest sensitivity (IC50 < 1 µg/ml) was demonstrated in four cell lines: KAI-3, NK-S1, NK-92, and KHYG-1 (IC50 36.0–303.6 ng/ml) (Fig. 1C, D; Additional file 1: Fig. S1), accompanied by an increase in sub-G1 fraction and S-phase arrest on cell cycle analyses (Additional file 1: Fig. S1C). In contrast, CDV and other selected antiviral agents (acyclovir, ganciclovir, adefovir, foscarnet, and penciclovir) did not show potent anti-lymphoma activity (Additional file 1: Fig. S2). Of note, the activity of BCV appeared to be independent of EBV positivity and was potent against the EBV-negative KHYH-1 cell line, though BCV was able to downregulate both EBNA1 and LMP1 protein expression levels in the EBV-positive KAI-3 and NK-S1 cell lines (Additional file 1: Fig. S3A, B). Intraperitoneal BCV (40 mg/kg, 2X per week) inhibited tumor growth in NOD/SCID mice NK-S1 xenografts, compared with vehicle alone (tumor volume: *p* = 0.0005; tumor weight: *p* = 0.0006) (Fig. 1E, F). No significant toxicity or body weight reduction was observed (Fig. 1G).

**Fig. 2:**
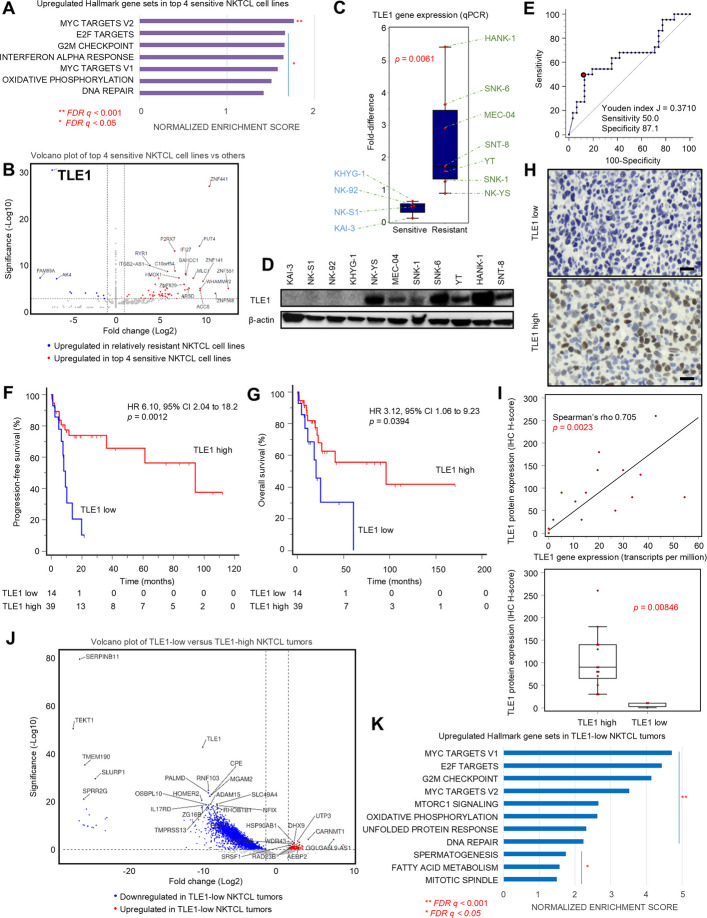
Differential gene expression pathways associated with BCV sensitivity in NK/T-cell lymphoma cell lines. **A** Gene set enrichment analysis showed that *MYC* target pathways and cell cycle-related pathways were prominently upregulated in the four sensitive cell lines compared to the rest. **FDR *q*-value < 0.001. *FDR *q*-value < 0.05. **B** Volcano plot showing the top differentially expressed genes between the top 4 sensitive NKTCL cell lines and the rest. *TLE1* was the top differentially expressed gene (log2foldchange − 7.39, adj. *p* < 0.0001). **C** *TLE1* gene expression was significantly higher in the more resistant NKTCL cell lines, validated by qPCR (*p* = 0.0061, Mann-Whitney *U*-test). **D** TLE1 protein expression was not detectable in the top 4 sensitive NKTCL cell lines. **E** In NKTCL patients, low *TLE1* gene expression (cutoff as determined by ROC analysis) was associated with **F** poorer progression-free survival and **G** overall survival. **H** Representative images of TLE1 IHC staining of NKTCL tumor specimens (scale bar: 20 µm). **I** TLE1 protein and gene expression are positively correlated (Spearman’s rho 0.705, *p* = 0.0023), and *TLE1* gene expression was significantly higher in TLE1-high cases (*n* = 13) compared with TLE1-low cases (*n* = 3) by protein expression (*p* = 0.00846, Mann-Whitney *U*-test). **J** Volcano plot showing the top differentially expressed genes between *TLE1*-high and *TLE1*-low NKTCL tumors. **K** Gene set enrichment analysis showed that *MYC* target pathways and cell cycle-related pathways were prominently upregulated in *TLE1*-low NKTCL tumor samples. **FDR *q*-value < 0.001. *FDR *q*-value < 0.05

RNAseq of all NKTCL cell lines and Hallmark GSEA demonstrated that *MYC* target pathways and cell cycle-related pathways (E2F targets, G2M checkpoint) were prominently upregulated in the four sensitive cell lines compared with the rest (FDR *q* < 0.01) (Fig. 2A and Additional file 2: Tables S1 and 2). Notably, *TLE1*, a known transcriptional repressor of the *MYC* oncogene, was the topmost downregulated gene (log2foldchange − 7.39, adjusted *p* < 0.0001) in the four BCV-sensitive cell lines. This finding was verified by qPCR (*p* = 0.0061) and by Western blot (Fig. 2B, C, D). Interestingly, patients with *TLE1*-low NKTCL had worse PFS (*HR* 6.10, 95% CI 2.04 to 18.2, *p* = 0.0012) and worse OS (*HR* 3.12, 95% CI 1.06 to 9.23, *p* = 0.0394) (Fig. 2E, F, G). TLE1 gene and protein expression were positively correlated, supporting potential ease of clinical testing via IHC (Fig. 2H, I). In keeping with results from cell lines, RNAseq of NKTCL patient samples (*n* = 53) showed that *TLE1*-low tumors were similarly enriched for genes involved in *MYC* target and cell cycle pathways (Fig. 2J, K and Additional file 2: Tables S3 and 4).

### Molecular pathways regulated by BCV in NKTCL

**Fig. 3:**
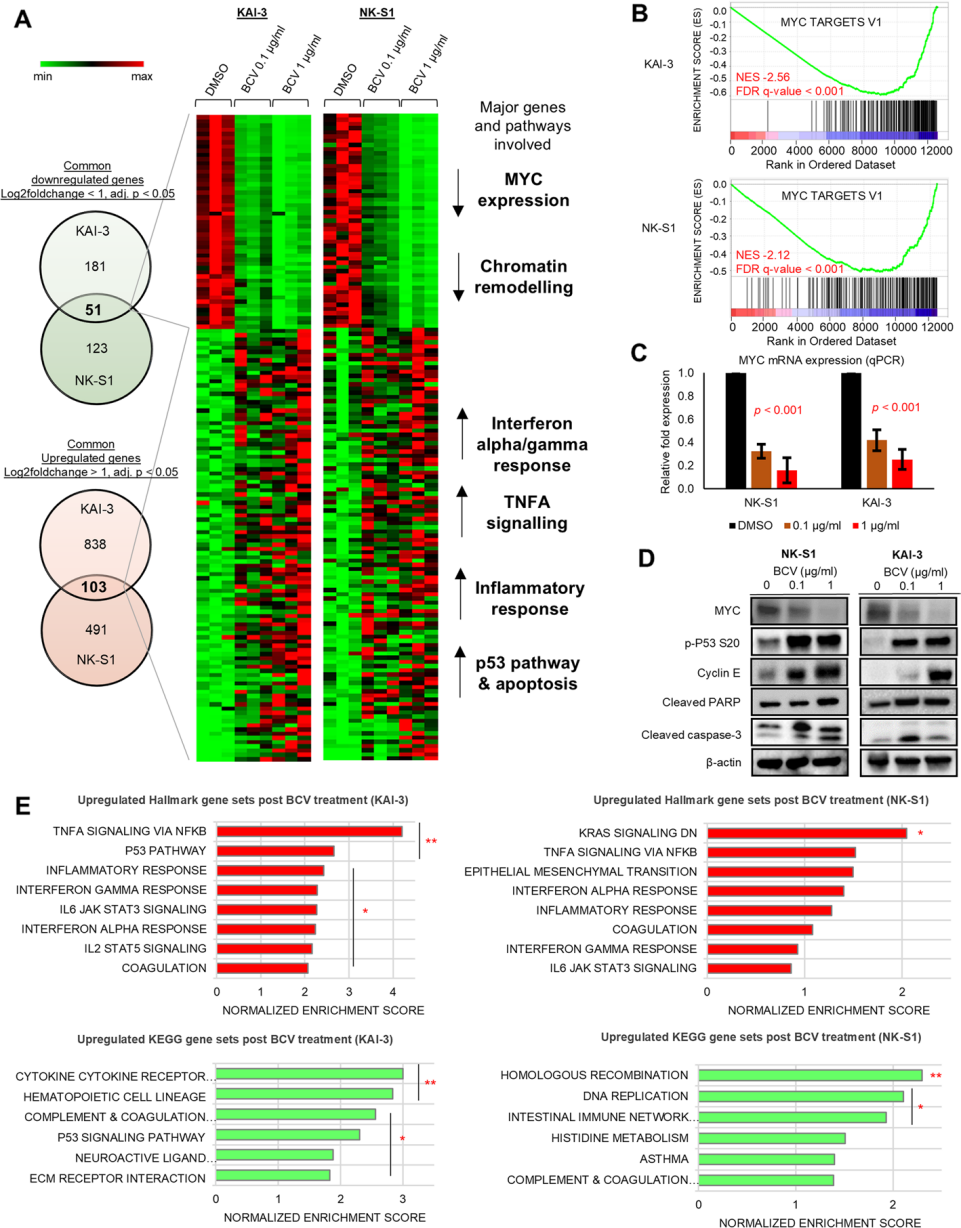
Brincidofovir inhibits MYC oncogenic signaling while activating DNA repair and immune-related pathways. **A** Whole transcriptomic sequencing of KAI-3 and NK-S1 cell lines treated with increasing doses of BCV (0.1 µg/ml and 1 µg/ml) revealed common downregulated (*n* = 51) and upregulated (*n* = 103) genes involved in various signaling pathways, based on MSigDB over-representation analysis (FDR *q* < 0.05). Data were obtained from three biological replicates. Scale bar showing min (0) and max (3006) gene counts in transcripts per million. **B** Gene set enrichment analysis showed that MYC targets were the top Hallmark gene sets downregulated in KAI-3 (MYC TARGETS V1: NES − 2.56, FDR *q* < 0.001) and NK-S1 (MYC TARGETS V1: NES =  − 2.12, FDR *q* < 0.001) post-BCV treatment. **C** Downregulation of *MYC* gene expression following BCV treatment in both KAI-3 and NK-S1 cell lines was validated by qPCR. Results shown are mean values and standard deviations from three independent experiments. **D** Western blot demonstrated decreased protein expression of MYC, while phopho-p53, cyclin E, cleaved PARP, and cleaved caspase-3 were increased. **E** Gene set enrichment analysis showed that immune-related, DNA replication and repair pathways were upregulated post-BCV treatment. **FDR *q*-value < 0.001. * FDR *q*-value < 0.05

In order to elucidate the molecular mechanisms underlying the anti-lymphoma effect of BCV, RNAseq and GSEA were performed on BCV-treated cell lines (KAI-3 and NK-S1). Common downregulated (*n* = 51) genes in both cell lines treated with increasing doses of BCV (0.1 µg/ml and 1 µg/ml) included *MYC*, as well as genes involved in chromatin remodeling. Common upregulated (*n* = 103) genes included those involved in various signaling pathways, such as interferon alpha and gamma response, TNFA signaling, inflammatory response, p53 pathway, and apoptosis. Notably, the top downregulated pathways on the KAI-3 and NK-S1 cell lines were both MYC targets V1 (normalized enrichment scores [NES] − 2.56 and − 2.12, respectively; both FDR *q*-value < 0.001) (Fig. 3A, B and Additional file 2: Tables S5 and 6). A dose-dependent downregulation of MYC expression by BCV was confirmed on qPCR and Western blot. BCV induced cyclin E and phospho-P53 (serine 20) expression, as well as cleavage of PARP and caspase-3, indicating S-phase arrest and programmed cell death by apoptosis. Similar results were observed in NK-92 and KHYG-1 cell-lines (Fig. 3C, D and Additional file 1: Fig. S3C). In keeping with these results, results from RNAseq and GSEA showed that immune-related, DNA replication and repair pathways were upregulated in KAI-3 and NK-S1 post-BCV treatment (Fig. 3E). Additionally, we investigated the effects of BCV on the JAK-STAT pathway, a key signaling pathway in NKTCL.^3^ BCV treatment led to a dose- and time-dependent decrease in total and phospho-STAT1, STAT3, and STAT5 protein expression in NK-S1 and KAI-3 cell lines (Additional file 1: Fig. S3D). ScRNAseq revealed distinct temporal cell states evoked by BCV treatment (1 µg/ml) for 48 h and 72 h in both KAI-3 and NK-S1 cell lines (Additional file 1: Fig. S4). In both cell lines, a significant shift in transcriptomic cell states occurred upon BCV treatment at 48 h and 72 h, with emergence of different cell clusters marked by perturbation of MYC and mTORC signaling, DNA repair, cell cycle and apoptosis pathways, and immune-mediated signals, among others (Additional file 2: Table S7).

### Immunogenic cell death response triggered by BCV

**Fig. 4:**
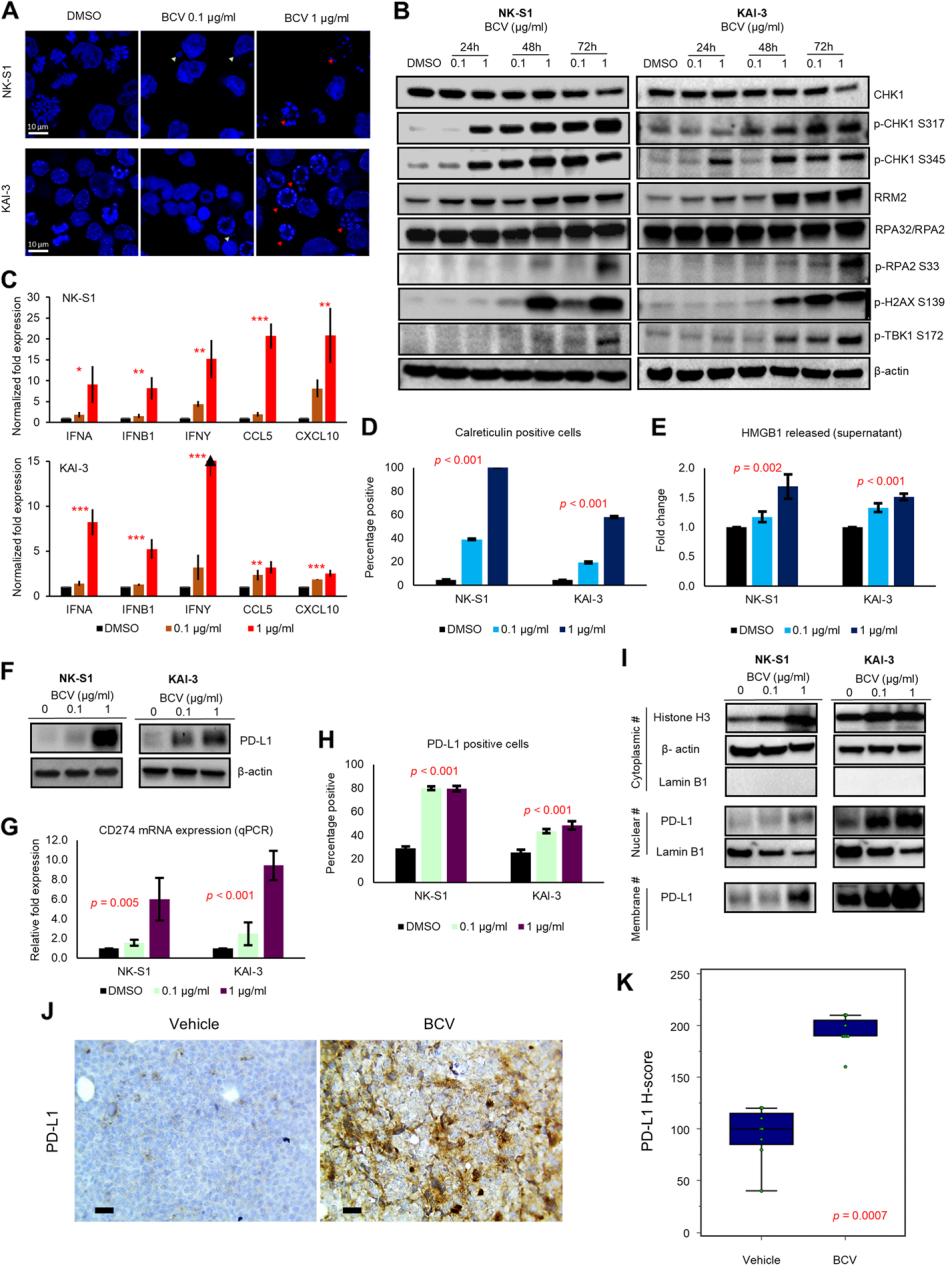
BCV triggers DNA damage, STING pathway activation, and immune signals in NKTCL cell lines. **A** Micronuclei formation and DNA fragmentation 48 h after BCV treatment (scale bar: 10 µm). **B** Western blot demonstrating dose- and time-dependent increase in replication stress markers, including p-CHK1 (S317 and S345), RRM2, and p-RPA2 (S33). Both p-H2AX (indicative of DNA double-strand breaks) and p-TBK1 (indicating STING pathway activation) are also upregulated in the same manner. **C** Upregulation of type I (IFNA, IFNB1) and II (IFNG) interferon and cytokine (CCL5, CXCL10) gene expression (qPCR) following BCV treatment (*p < 0.05, **p < 0.01, ***p < 0.001, one-way ANOVA). **D** Increase in the proportion of calreticulin-expressing cells and **E** the extracellular release of HMGB1 upon BCV treatment. **F** Upregulation of PD-L1 protein expression by Western blot, **G** CD274 gene expression by qPCR, and **H** PD-L1-expressing cells by flow cytometry. The qPCR, flow cytometry, and HMGB1 release assay data shown are mean values and standard deviations from three independent experiments. **I** BCV treatment increased cytoplasmic DNA levels (indicated by histone H3). PD-L1 protein levels were upregulated in both membranous and nuclear fractions. **J** Representative images of PD-L1 IHC on NK-S1 xenograft tumors in vehicle and BCV-treated mice (scale bar: 20 µm). **K** IHC on NK-S1 xenograft tumors showed increased protein expression of PD-L1 in BCV-treated mice (*n* = 8) compared with vehicle-treated controls (*n* = 8) (*p* = 0.0007, Mann-Whitney *U*-test)

To further investigate the cell death response evoked by BCV, we performed confocal microscopy and showed that BCV triggered micronuclei formation and DNA fragmentation in both KAI-3 and NK-S1 cell lines (Fig. 4A). The Western blot demonstrated an increase in replication stress markers (p-CHK1 S317 and S345, RRM2, p-RPA2 S33), as well as p-H2AX (DNA double-strand break) and p-TBK1 (STING pathway activation) (Fig. 4B). Gene expression levels of type I (*IFNA*, *IFNB1*) and II (*IFNG*) interferons and cytokines (*CCL5*, *CXCL10*) were upregulated following BCV treatment, as did the proportion of surface calreticulin-expressing cells and the amount of extracellular HMGB1 released, indicative of immunogenic cell death (IMCD) (Fig. 4C, D, E). As a potential predictive biomarker of immunotherapy response [16], we also observed a modest increase in IFN-γ protein expression (Additional file 1: Figs. S3E and S12). Notably, the immune checkpoint PD-L1 gene (*CD274*) and protein expression were increased following BCV treatment (Fig. 4F, G), the latter including both nuclear and membranous protein compartments (Fig. 4H, I and Additional file 1: Fig. S12). There was a significant increase in cytoplasmic histone H3 protein expression following BCV treatment, indicative of cytoplasmic DNA release (Fig. 4I). The in vivo response to BCV treatment on NK-S1 xenograft tumors similarly showed increased protein expression of PD-L1 in BCV-treated mice (*n* = 8) compared with vehicle-treated controls (*n* = 8), with a 1.9-fold higher median H-score (*p* = 0.0007) (Fig. 4J, K). In NK-92 and KHYG-1 cell lines, total and surface PD-L1 protein expression, as well as surface calreticulin expression, were similarly increased following BCV treatment (Additional file 1: Fig. S3F, G, H).

### In vitro and in vivo activity of BCV in PTCL

**Fig. 5:**
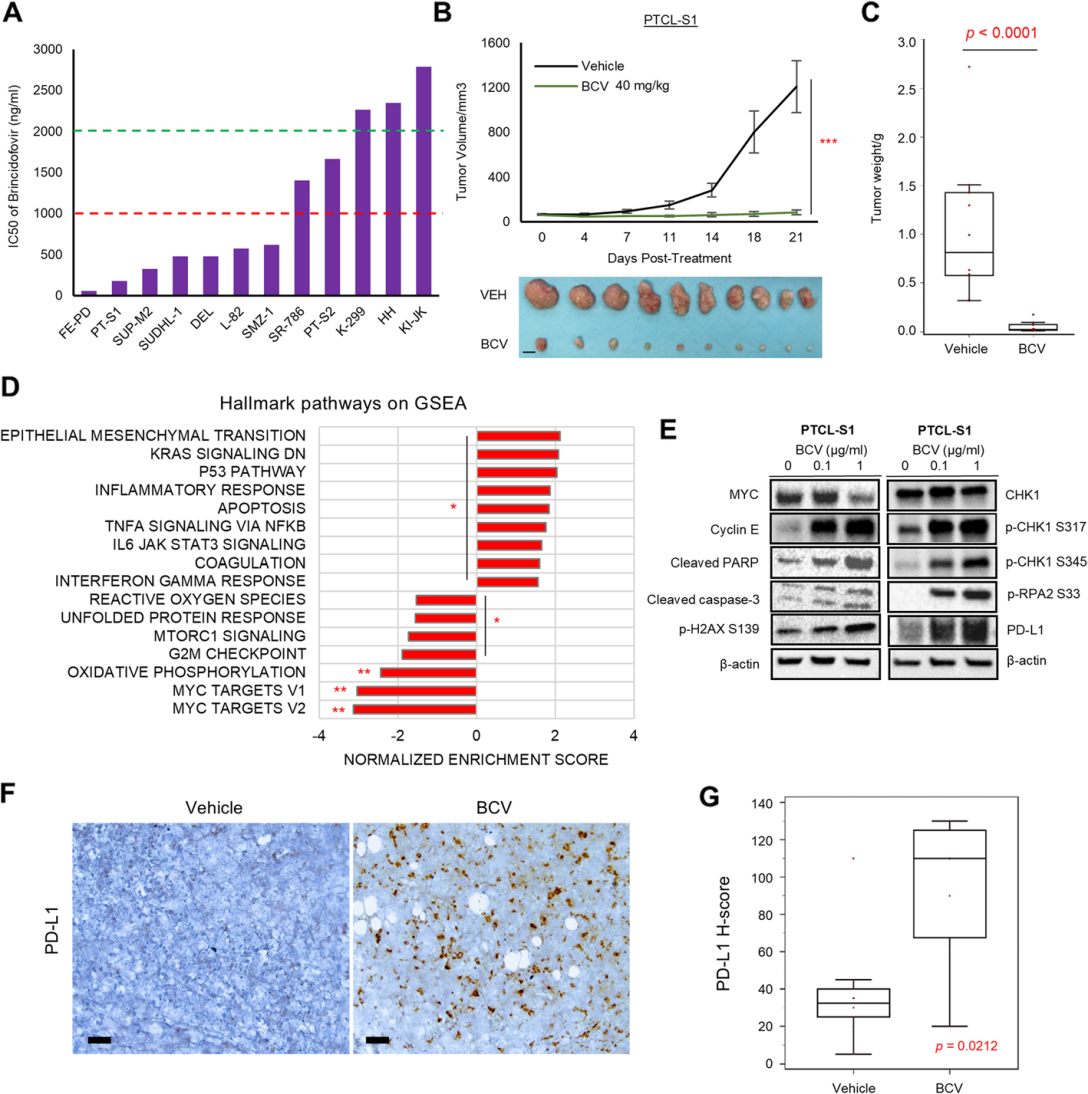
Preclinical efficacy of brincidofovir in peripheral T-cell lymphoma. **A** IC50 of BCV across various T-cell lymphoma cell lines. **B** BCV inhibited tumor growth in the PTCL-S1 xenograft model (tumor volume: *p* < 0.0001, two-tailed *t*-test). **C** Tumor weight: *p* < 0.0001, Mann-Whitney *U*-test. NSG mice were treated twice per week IP with either vehicle or BCV (40 mg/kg) starting 7 days after subcutaneous flank inoculation with 0.5 × 10^6^ PTCL-S1 cells (*n* = 10 per group) (scale bar: 10 mm). **D** Gene set enrichment analysis showed that several pathways were deregulated in PTCL-S1 post-BCV treatment, including downregulation of MYC target pathways. ** FDR *q*-value < 0.001. *FDR *q*-value < 0.05. **B** BCV inhibited tumor growth in the PTCL-S1 xenograft model (tumor volume: *p* < 0.0001, two-tailed *t*-test). **C** Tumor weight: *p* < 0.0001, Mann-Whitney *U*-test. NSG mice were treated twice per week IP with either vehicle or BCV (40 mg/kg) starting 7 days after subcutaneous flank inoculation with 0.5 × 10^6^ PTCL-S1 cells (*n* = 10 per group) (scale bar: 10 mm). **D** Gene set enrichment analysis showed that several pathways were deregulated in PTCL-S1 post-BCV treatment, including downregulation of MYC target pathways. ** FDR *q*-value < 0.001. *FDR *q*-value < 0.05. **E** Western blot demonstrated decreased protein expression of MYC, while cyclin E, cleaved PARP, and cleaved caspase-3 were increased. p-H2AX, p-CHK1, p-RPA2, and PD-L1 were similarly increased. **F** Representative images of PD-L1 IHC on PTCL-S1 xenograft tumors in vehicle and BCV-treated mice (scale bar: 50 µm). **G** IHC on PTCL-S1 xenograft tumors showed increased protein expression of PD-L1 in BCV-treated mice (*n* = 7) compared with vehicle-treated controls (*n* = 10) (*p* = 0.0212, Mann-Whitney *U*-test)

**Fig. 6:**
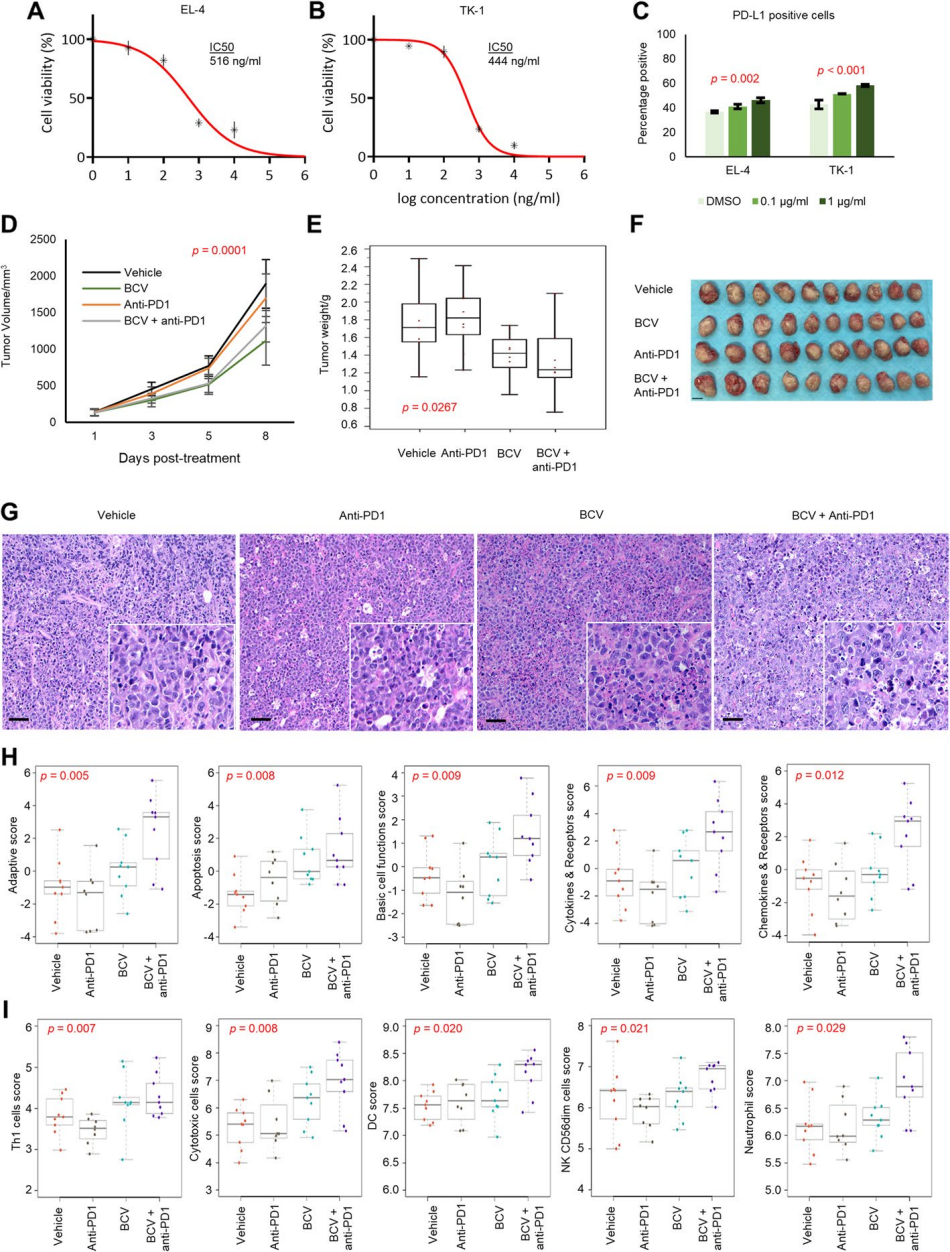
Combination of BCV and anti-PD1 immune checkpoint blockade. **A**, **B** BCV inhibits cell growth of murine T-cell lymphoma cell lines EL-4 and TK-1 in a dose-dependent manner. Drug treatments were performed in triplicate, and results are represented by mean values and standard deviations. **C** BCV increased PD-L1 expression in both murine T-cell lymphoma cell lines by flow cytometry. **D**, **E**, **F** BCV inhibited tumor growth in the EL-4 xenograft model in C57BL/6J mice (tumor volume: *p* = 0.0001, two-tailed *t*-test; tumor weight: *p* = 0.0267, Mann-Whitney *U*-test). BCV alone (IP 40 mg/kg) or in combination with anti-PD1 (IP 200 µg 1 × per week) significantly inhibited tumor growth at 8 days post-treatment compared with isotype or anti-PD1 treatment alone. No significant difference in tumor volume or weight was observed with the combination of BCV and anti-PD1 compared with BCV alone. Subcutaneous flank inoculation was performed with 0.5 × 10^6^ EL-4 cells (*n* = 10 per group) (scale bar: 10 mm). **G** Representative H&E images showing increased apoptosis in BCV-treated tumors and brisk immune infiltration in the BCV + anti-PD1 combination group, relative to non-BCV-treated groups (20 × magnification; scale bar: 50 µm; inset shows 40 × magnification). **H** NanoString Mouse Immunology Panel demonstrated the highest scores for adaptive immune response, apoptosis, basic cell functions, cytokines/chemokines, and receptors pathways in the combination group. **I** Cell types significantly enriched in the BCV + anti-PD1 combination group are shown

Next, BCV was investigated in a cohort of T-cell lymphoma cell lines (anaplastic large cell lymphoma [ALCL], *n* = 9; PTCL not otherwise specified [PTCL-NOS], *n* = 2; primary cutaneous T-cell lymphoma [CTCL], *n* = 1). BCV inhibited viability across all T-cell lymphoma cell lines in a dose-dependent manner (median IC50 = 593 ng/ml; range, 60.2 to 2785 ng/ml). Marked sensitivity (*IC50* < 1000 ng/ml) was demonstrated in most cell lines (7 of 11), including our in-house PTCL-S1 (*TP63*-rearranged ALCL) and PTCL-S2 (PTCL-NOS) cell-line models (*IC50* = 177 ng/ml and 1664 ng/ml, respectively) (Fig. 5A and Additional file 1: Fig. S5) [17]. In vivo, intraperitoneal BCV (40 mg/kg, 2 × per week) inhibited tumor growth in both NOD/SCID mice PTCL-S1 and PTCL-S2 xenografts, compared with vehicle alone. For PTCL-S1, at 21-day post-treatment with BCV, tumor volume was significantly reduced (81.9 mm^3^ vs 1207.1 mm^3^, *p* < 0.0001), as was tumor weight (0.021 g vs vehicle: 0.812 g, *p* < 0.0001) (Fig. 5B, C). For PTCL-S2, both tumor volume (*p* = 0.0113) and tumor weight (*p* = 0.0039) were both significantly reduced with BCV treatment as compared to vehicle control (Fig. 6SA, B). Like in NKTCL, RNAseq and GSEA showed that MYC target and cell cycle pathways were significantly downregulated in PTCL-S1 cell-line post-BCV treatment. Upregulation of interferon (*IFNB1*, *IFNG*), cytokine (*CCL5*, *CXCL10*), and *CD274* gene expression was observed. Western blot analysis showed downregulation of MYC protein and upregulation of apoptosis markers (cleaved PARP, cleaved caspase-3), phospho-H2AX, phospho-CHK1 (Ser317/Ser345), p-RPA2 (Ser33), and PDL1 in BCV-treated PTCL-S1 and PTCL-S2 cell-lines (Fig. 5D, E and Additional file 1: Fig. S6C; Additional file 2: Table S8). BCV treatment on PTCL-S1 xenograft tumors similarly showed increased protein expression of PD-L1 in BCV-treated mice (*n* = 7) compared with vehicle-treated controls (*n* = 10) (*p* = 0.0212) (Fig. 5F, G).

### Immune response activation by BCV and anti-PD1 immune checkpoint blockade

In order to investigate the in vivo immune response further, we first examined the effect of BCV in murine lymphoma cell lines EL-4 and TK-1, demonstrating a dose-dependent inhibition of cell viability in both models (IC50 516 ng/ml and 444 ng/ml, respectively), as well as increased surface expression of PD-L1 (Fig. 6A, B, C). Deploying a syngeneic EL4-C57BL/6 model, BCV alone or in combination with anti-PD1 (intraperitoneal 200 µg 2 × per week) significantly inhibited tumor growth at 8 days post-treatment, compared to isotype or anti-PD1 treatment alone. However, no significant difference in tumor volume or weight was observed with the combination of BCV and anti-PD1, compared with BCV alone (Fig. 6D, E, F). Intriguingly, at this early time point, examination of H&E staining showed a significant immune response in BCV-treated groups, which appeared particularly brisk with the addition of anti-PD1 (Fig. 6G). NanoString profiling showed a significantly heavier immune infiltration in the BCV + anti-PD1 combination group, as corroborated by the highest scores for adaptive immune response, cytokines/chemokines and receptors, cytotoxic cells, dendritic cells, NK CD56^dim^ cells, and neutrophils. Gene expression of *CCL2*, *CCL12*, *CXCL9*, *GZMB*, *CHIL3*, and *CTLA4* was also highest in the combination treatment group (Fig. 6H, I and Additional file 1: Fig. S7, 8, 9; Additional file 2: Tables S9 and 10).

### Extending the preclinical activity of BCV to B-cell lymphoma models

**Fig. 7:**
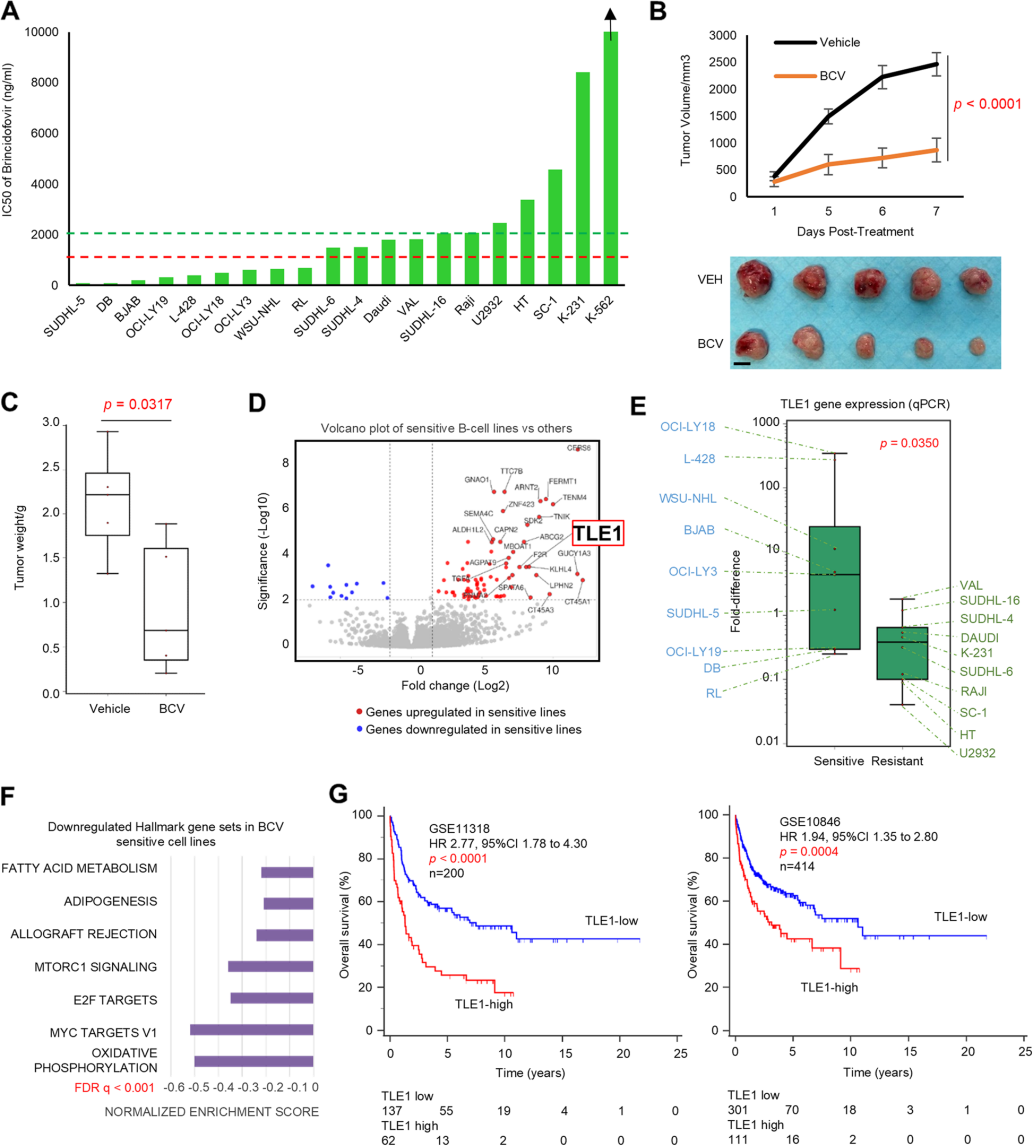
Preclinical efficacy of brincidofovir in B-cell lymphoma. **A** IC50 of BCV across B-cell lymphoma cell lines. **B**, **C** BCV inhibited tumor growth in the OCI-LY18 xenograft model (tumor volume: *p* < 0.0001, two-tailed *t*-test; tumor weight: *p* = 0.0317, Mann-Whitney *U*-test). NSG mice were treated twice per week IP with either vehicle or BCV (40 mg/kg) following flank inoculation with 0.5 × 10^6^ OCI-LY18 cells (*n* = 5 per group) (scale bar: 10 mm). **D** Volcano plot showing the top differentially expressed genes between the top sensitive B-cell lymphoma cell lines (*n* = 9) and the rest. TLE was among the top differentially expressed genes (log2foldchange 8.12, adj. *p* = 0.0004). **E** *TLE1* gene expression was significantly higher in the more sensitive B-cell lymphoma cell lines, validated by qPCR (*p* = 0.0350). **F** Gene set enrichment analysis showed that oxidative phosphorylation, *MYC* target, and E2F target pathways were downregulated in the sensitive cell lines compared with the rest. **G** In DLBCL patients, high *TLE1* gene expression was associated with poorer progression-free survival and overall survival

Finally, BCV was investigated in a cohort of B-cell lymphoma cell lines. BCV inhibited viability in all B-cell lymphoma cell lines in a dose-dependent manner (IC50 79.8 to 8414 ng/ml). Marked sensitivity (IC50 < 1 µg/ml) was demonstrated in nine cell lines, including the *MYC*/*BCL2*-rearranged double-hit DLBCL cell-line OCI-LY18 (IC50: 500 ng/ml) (Fig. 7A and Additional file 1: Fig. S10). Intraperitoneal BCV (40 mg/kg, 2 × per week) inhibited tumor growth in NOD/SCID mice bearing OCI-LY18 xenografts, compared with vehicle alone (tumor volume: *p* < 0.0001; tumor weight: *p* = 0.0317) (Fig. 7B, C). Interestingly, in contrast to T and NK/T cell lines, RNAseq showed that the transcriptional repressor *TLE1* was instead among the top upregulated genes in the BCV-sensitive cell lines (IC50 < 1 µg/ml) (log2foldchange = 8.12, adjusted *p* = 0.0004), which was validated by qPCR (*p* = 0.0350) (Fig. 7D, E and Additional file 2: Table S11). Gene set enrichment analysis showed that oxidative phosphorylation, MYC target, and E2F target pathways were downregulated in the sensitive cell lines compared to the rest (Fig. 7F). DLBCL patient samples from two independent datasets showed that *TLE1*-high tumors conferred worse OS (GSE11318, *n* = 200: *HR* 2.77, 95% CI 1.78–4.30, *p* < 0.0001; GSE10846, *n* = 414: *HR* 1.94, 95% CI 1.35–2.80, *p* = 0.0004). *TLE1* expression level was found to be an independent predictor for OS in one dataset (GSE11318) [18] but not the other (GSE10846) [19], and the prognostic implications of *TLE1* expression will need to be validated in prospective datasets (Fig. 7G and Additional file 2: Table S12).

## Discussion

In this study, we investigated the preclinical therapeutic activity of the novel nucleoside analogue BCV in a spectrum of non-Hodgkin lymphoma cell lines. Originally intended as an antiviral agent against smallpox and other viruses, our observations in vitro and in vivo support its potential role as an anticancer agent, regardless of the presence of any known associated viruses. Furthermore, by examining transcriptomic profiles of the cell line models, we identified potential signaling pathways modulating the response to BCV, including STING pathway activation and PD-L1 expression, implying prospective synergies with checkpoint immunotherapy.

Previous studies on CDV have demonstrated growth inhibitory effects in various cancer types, including viral-negative tumors such as melanoma [20], glioblastoma [21], and squamous carcinoma of the head and neck (HNSCC) and of the cervix [9, 10]. In a vascular tumor model induced by basic fibroblast growth factor (FGF2)-overexpressing endothelial cells (FGF2-T-MAE) in mice, CDV evoked S-phase cell cycle arrest and apoptosis [22]. In glioblastoma, the cytotoxic effect of CDV was mediated through apoptosis, regardless of the presence of associated cytomegalovirus infection. In this study, CDV was shown to incorporate into tumor cell DNA, thereby promoting replication fork stalling and initiating the formation of DNA double-strand breaks [21]. Similar findings were also observed in HNSCC and cervical carcinoma [9, 10]. In keeping with these earlier studies, our current results support a similar mechanism of action for BCV in PTCL and NKTCL, in which BCV triggers replication stress and cell cycle arrest, as well as DNA damage and apoptosis.

The cyclic GMP–AMP synthase (cGAS) stimulator of interferon genes (STING) pathway is a key mediator of the inflammatory response to DNA damage, including that induced by some exogenous anti-tumor agents [11]. BCV exposure leads to accumulation of cytosolic DNA, cGAS/STING pathway activation, triggering an interferon response and upregulation of the PD-L1 immune checkpoint protein. Of note, interferons have been shown to be potent inducers of PD-L1 [23]. In addition, BCV evokes IMCD, as evidenced by increased cell membranous expression of calreticulin, a danger-associated molecular pattern (DAMP) molecule, where it acts as an “eat me” signal for immature dendritic cells [24]. An increase in secreted high mobility group box 1 (HMGB1) protein levels was also observed, which is responsible for the optimal release and presentation of tumor antigens to dendritic cells, dendritic cell maturation, and synthesis of proinflammatory molecules [25, 26]. Indeed, our results using a syngeneic EL4-C57BL/6 lymphoma model further suggest a robust local inflammatory response in response to the addition of PD1 blockade to BCV treatment, including activation of adaptive immune responses, cytokines/chemokines and receptors, cytotoxic cells, dendritic cells, NK CD56^dim^ cells, and neutrophils. The potential for combination with immune checkpoint blockade, as well as with other DNA-damaging agents or radiation therapy, deserves to be evaluated in further studies.

TLE1 functions as a transcriptional corepressor that can antagonize the functions of a number of transcription factors involved in key signals mediating oncogenesis and inflammation, including Notch, Wnt, and NF-κB pathways [27]. In a prior study by Fraga et al., TLE1 was found to be epigenetically inactivated in various hematologic malignancies through CpG island promoter hypermethylation. Reintroduction of TLE1 caused growth inhibition, whereas depletion resulted in growth enhancement, supporting its role as a tumor suppressor [28]. Our results showed that in NKTCL cell lines, *TLE1* gene expression was inversely correlated with sensitivity to BCV. *TLE1*-low tumors were enriched for *MYC* target pathways, which were prominently downregulated by BCV treatment. In patients with NKTCL, we further demonstrated that low tumor levels of *TLE1* gene expression were associated with worse survival outcomes. Previously, in patients with T-cell acute lymphoblastic leukemia (T-ALL), low *TLE1* expression was similarly associated with a higher relapse rate and shorter survival [29, 30]. Taken together, this implies that *TLE1* gene expression may be both a potential predictive and prognostic biomarker in NKTCL, and that patients with low tumor *TLE1* expression might derive greater benefit from BCV therapy. Interestingly, however, our results demonstrated the converse instead for B-cell lymphoma — *TLE1* was significantly upregulated in the cell lines that were more sensitive to BCV, and high *TLE1* gene expression instead conferred poor survival in patients with DLBCL. The specific mechanisms of TLE1 function might be contextual to the hematopoietic lineage and will require further elucidation in future studies.

Our work demonstrates the therapeutic potential of BCV in non-Hodgkin lymphoma, regardless of the presence of EBV. While our study remains limited to a preclinical investigation of BCV, remarkable in vivo anti-tumor activity was demonstrated in classically aggressive forms of lymphoma, including NKTCL, ALK-negative ALCL, and double-hit DLBCL xenograft models. We have also gained insight into potential mechanisms of anti-tumor action of BCV, including its ability to evoke immunogenic cell death. Supporting the feasibility of repurposing BCV for lymphoma treatment, data obtained from previous clinical trials support the practicability of attaining a plasma concentration of more than 2000 ng/ml while avoiding severe treatment-related adverse effects [31–33].

## Conclusions

In conclusion, we propose that BCV is an attractive agent for the treatment of non-Hodgkin lymphoma, particularly PTCL and NKTCL. A clinical trial (NCT06761677) is underway to confirm its safety and efficacy in patients with relapsed and/or refractory non-Hodgkin lymphoma. Further translational studies will be required to validate predictive biomarkers of response.

## Supplementary Information

Additional file 1. Figs. S1–S12. Supplementary Methods. Fig. S1. Effect of BCV in NKTCL cell lines. Fig. S2. Activity of selected antivirals in NKTCL cell lines. Fig. S3. Downstream mechanisms of BCV in NKTCL cell lines. Fig. S4. Single cell transcriptomic sequencing reveals distinct cell states evoked by BCV treatment. Fig. S5. Effect of BCV in T-cell lymphoma cell lines. Fig. S6. In vivo efficacy of BCV in PTCL-S2 xenograft model. Fig. S7. NanoString pathway scores in EL4-C57BL/6 treatment groups. Fig. S8. NanoString cell-type scores and expression of selected genes in EL4-C57BL/6 treatment groups. Fig. S9. Selected validation data for NanoString profiling results of EL4-C57BL/6 treatment groups. Fig. S10. Effect of BCV in B-cell lymphoma cell lines. Fig. S11. Expanded Western blot images used in the manuscript. Fig. S12. FACS gating strategies. Supplementary Methods. SDS-PAGE and Western blot; Quantification of gene expression via quantitative polymerase chain reaction (qPCR); Immunohistochemistry; Confocal microscopy and identification of micronuclei; Cell cycle analysis; Flow cytometry for IFN-γ, calreticulin and PD-L1 expression; HMGB1 release assay; NanoString gene expression profiling.

Additional file 2. Tables S1–S14. Table S1. Differentially regulated genes in sensitive versus resistant NKTCL cell lines. Table S2. Differentially regulated pathways in sensitive versus resistant NKTCL cell lines. Table S3. Clinicopathological features of the patient cohort with NKTCL. Table S4. Differentially regulated pathways in TLE1-low and TLE1-high NKTCL tumor samples. Table S5. Differentially regulated pathways in NK-S1 cell line following BCV treatment. Table S6. Differentially regulated pathways in KAI-3 cell line following BCV treatment. Table S7. Differentially regulated pathways from scRNAseq clusters in KAI-3 and NK-S1 cell-lines following BCV treatment. Table S8. Differentially regulated pathways in PTCL-S1 cell line following BCV treatment. Table S9. NanoString pathway scores in EL4-C57BL/6 treatment groups. Table S10. NanoString cell-type scores in EL4-C57BL/6 treatment groups. Table S11. Differentially regulated genes in sensitive versus resistant B-cell lymphoma cell lines. Table S12. Multivariate analysis of TLE1 gene expression with overall survival in DLBCL datasets. Table S13. List of cell-line models used in the study. Table S14. List of antibodies and primers used in the study.

## Data Availability

Gene expression data and single cell transcriptomic data have been deposited in Gene Expression Omnibus (accession numbers GSE293367, GSE295559, GSE295560, GSE295561, GSE295562, GSE295563, GSE295564).

## Abbreviations

BCV: Brincidofovir
T/NK-NHL: T/NK-cell non-Hodgkin lymphoma
BCL: B-cell lymphoma
PTCL: Peripheral T-cell lymphoma
NKTCL: Natural killer/T-cell lymphoma
CDV: Cidofovir
EBV: Epstein-Barr virus
FFPE: Formalin-fixed paraffin-embedded
IACUC: Institutional Animal Care and Use Committee
GSEA: Gene set enrichment analysis
ScRNAseq: Single-cell RNA-seq
GEMs: Gel beads in emulsion
UMI: Unique molecular index
3′GEX: 3′ Gene expression
ROC: Receiver operative characteristic
OS: Overall survival
PFS: Progression-free survival
NES: Normalized enrichment score
IMCD: Immunogenic cell death
ALCL: Anaplastic large cell lymphoma
PTCL-NOS: PTCL not otherwise specified
CTCL: Primary cutaneous T-cell lymphoma
cGAS: Cyclic GMP–AMP synthase
STING: Stimulator of interferon genes
DAMP: Danger-associated molecular pattern
HMGB1: High mobility group box 1
T-ALL: T-cell acute lymphoblastic leukemia
