# Supplementary material for: Preclinical activity of brincidofovir in peripheral T-cell and NK/T-cell lymphoma
Source: BMC Med. 2026 Feb 6;24:147. doi: 10.1186/s12916-026-04680-8 (PMC12973774; doi:10.1186/s12916-026-04680-8)
Supplement: Supplementary file 1 — Additional file 1. Figs. S1–S12. Supplementary Methods. Fig. S1. Effect of BCV in NKTCL cell lines. Fig. S2. Activity of selected antivirals in NKTCL cell lines. Fig. S3. Downstream mechanisms of BCV in NKTCL cell lines. Fig. S4. Single cell transcriptomic sequencing reveals distinct cell states evoked by BCV treatment. Fig. S5. Effect of BCV in T-cell lymphoma cell lines. Fig. S6. In vivo efficacy of BCV in PTCL-S2 xenograft model. Fig. S7. NanoString pathway scores in EL4-C57BL/6 treatment groups. Fig. S8. NanoString cell-type scores and expression of selected genes in EL4-C57BL/6 treatment groups. Fig. S9. Selected validation data for NanoString profiling results of EL4-C57BL/6 treatment groups. Fig. S10. Effect of BCV in B-cell lymphoma cell lines. Fig. S11. Expanded Western blot images used in the manuscript. Fig. S12. FACS gating strategies. Supplementary Methods. SDS-PAGE and Western blot; Quantification of gene expression via quantitative polymerase chain reaction (qPCR); Immunohistochemistry; Confocal microscopy and identification of micronuclei; Cell cycle analysis; Flow cytometry for IFN-γ, calreticulin and PD-L1 expression; HMGB1 release assay; NanoString gene expression profiling. [file 12916_2026_4680_MOESM1_ESM.docx]

**SUPPLEMENTAL DATA**

**CONTENTS**

**1.0 Supplementary Figures**

Figure S1. Effect of BCV in NKTCL cell lines

Figure S2. Activity of selected antivirals in NKTCL cell lines

Figure S3. Downstream mechanisms of BCV in NKTCL cell lines

Figure S4. Single cell transcriptomic sequencing reveals distinct cell states evoked by BCV treatment

Figure S5. Effect of BCV in T-cell lymphoma cell lines

Figure S6. In vivo efficacy of BCV in PTCL-S2 xenograft model

Figure S7. NanoString pathway scores in EL4-C57BL/6 treatment groups

Figure S8. NanoString cell-type scores and expression of selected genes in EL4-C57BL/6 treatment groups

Figure S9. Selected validation data for NanoString profiling results of EL4-C57BL/6 treatment groups

Figure S10. Effect of BCV in B-cell lymphoma cell lines

Figure S11. Expanded Western blot images used in the manuscript

Figure S12. FACS gating strategies

**2.0 Supplementary Methods**


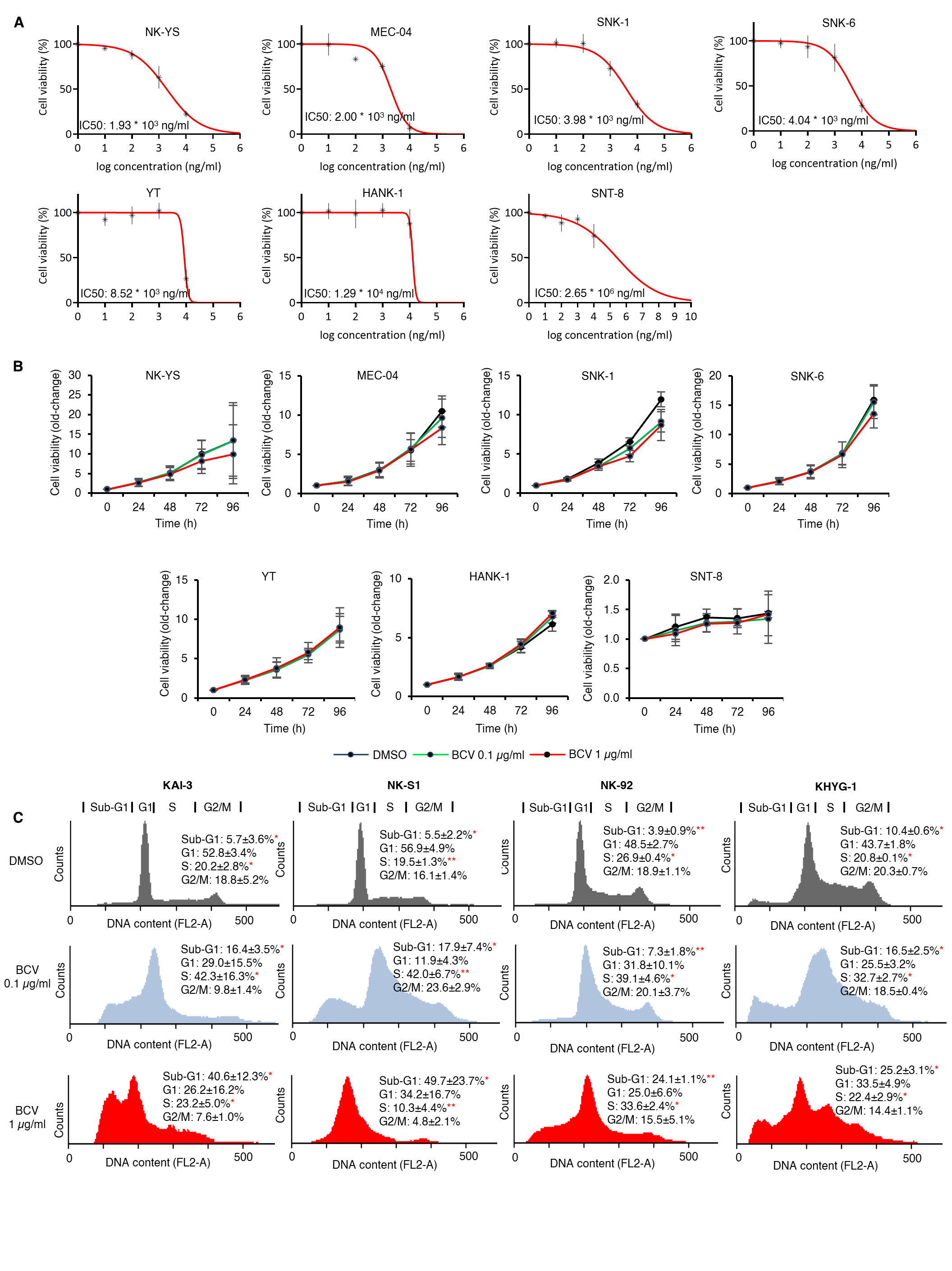


**Figure S1. Effect of BCV in NKTCL cell lines**

(A) Dose response curves of NKTCL cell lines following BCV treatment.

(B) Time response curves of cell viability following BCV treatment in NKTCL cell lines.

(C) BCV evoked a dose-dependent increase in the sub-G1 cell fraction and induced S-phase arrest in the 4 most sensitive cell lines tested. **p* < 0.05 ***p* < 0.001


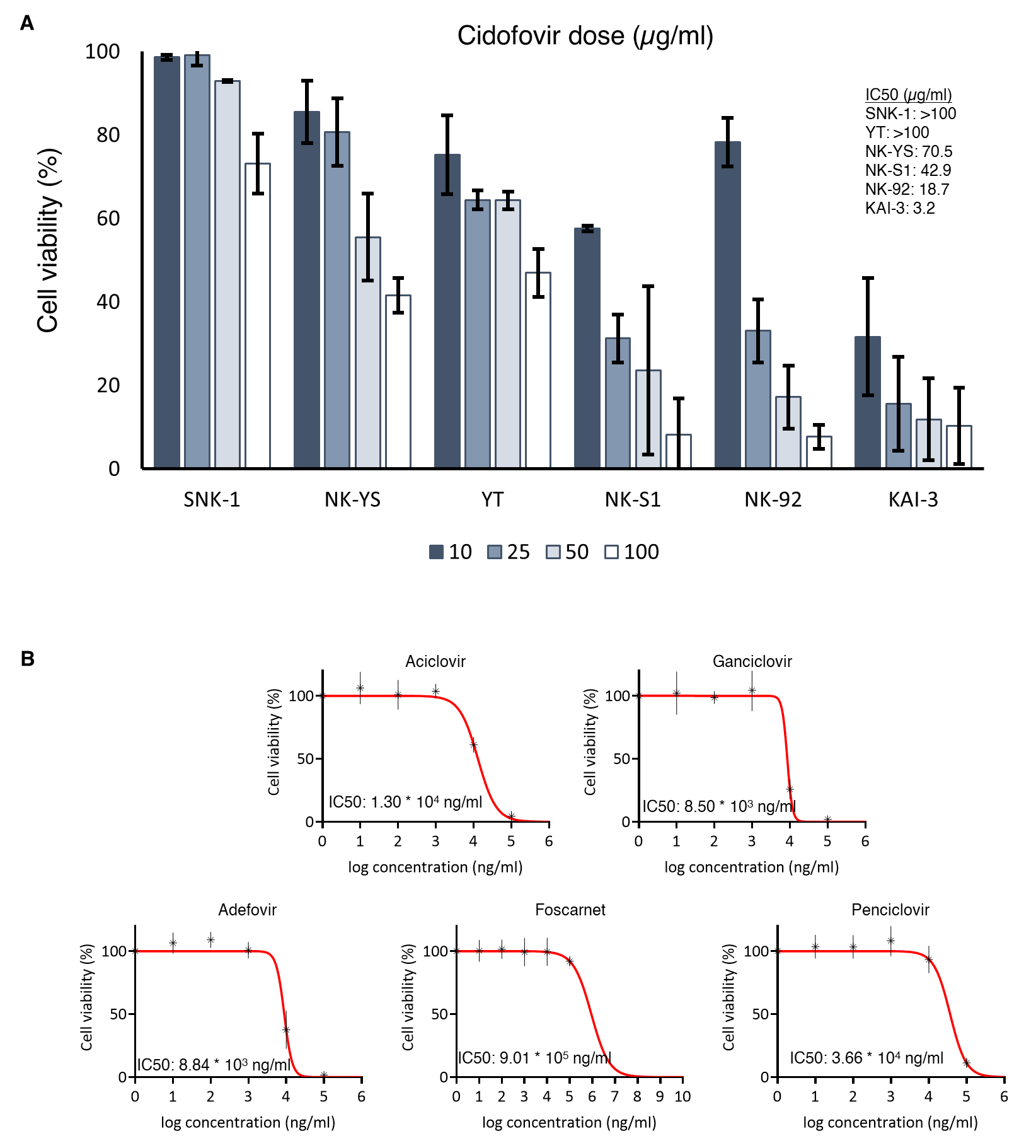


**Figure S2. Activity of selected antivirals in NKTCL cell lines**

(A) Effect of cidofovir on cell viability in NKTCL cell lines.

(B) Effect of selected antiviral drugs in the NK-S1 cell line.


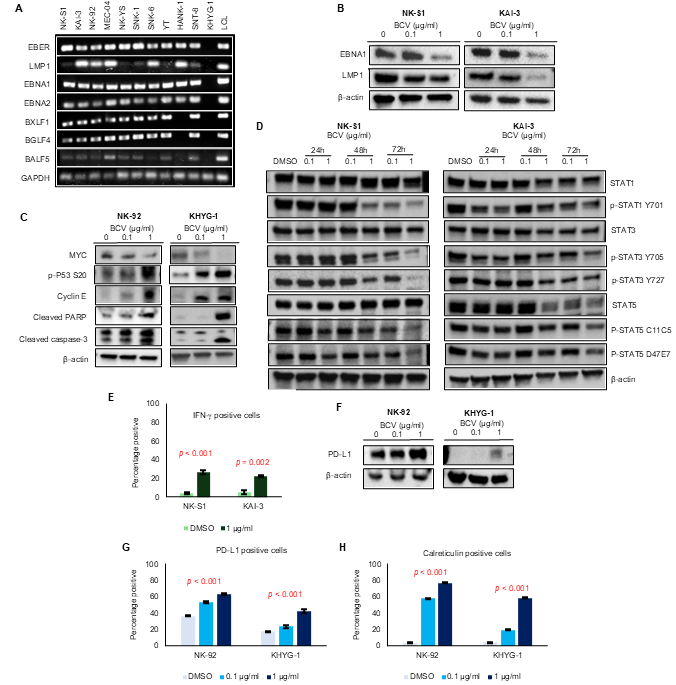


**Figure S3. Downstream mechanisms of BCV in NKTCL cell lines**

(A) Variable expression of EBV transcripts across NKTCL cell lines, including both latent genes *LMP1*, *EBNA1*, *EBNA2*, as well as lytic genes *BXLF1*, *BGLF4* and *BALF5*. Included controls are EBV-negative NKTCL cell line KHYG-1 and EBV-immortalized lymphoblastoid cell line (LCL).

(B) BCV downregulates EBNA1 and LMP1 protein expression in NK-S1 and KAI-3 cell lines.

(C) Decreased protein expression of MYC, and increased phopho-p53, cyclin E, cleaved PARP and cleaved caspase-3 protein expression in NK-92 and KHYG-1 cell lines.

(D) BCV treatment led to a dose and time-dependent decrease in total and phospho-STAT1, STAT3, and STAT5 protein expression in NK-S1 and KAI-3 cell lines.

(E) Increased in IFN-γ positive cells on flow cytometry in NK-S1 and KAI-3 cell lines.

(F) Upregulation of PD-L1 protein expression on Western blot and (G) PD-L1-expressing cells on flow cytometry in NK-92 and KHYG-1 cell lines.

(H) Increase in proportion of calreticulin-expressing cells upon BCV treatment.


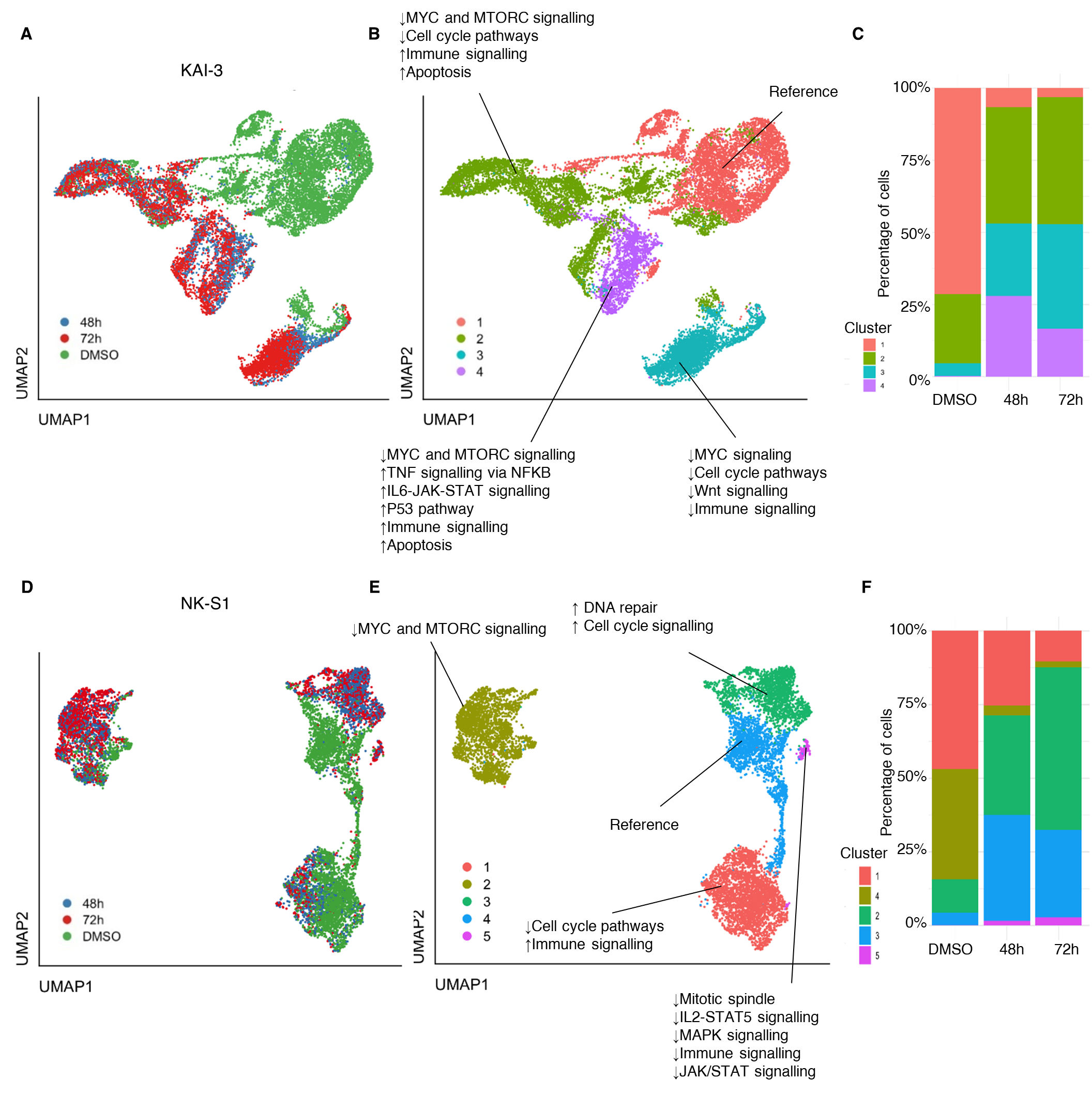


**Figure S4. Single cell transcriptomic sequencing reveals distinct cell states evoked by BCV treatment**

(A) UMAP plots demonstrating distinct clusters of mock (DMSO)-treated cells and BCV-treated cells at 48h and 72h for KAI-3 cell line.

(B) UMAP plots demonstrated distinct clusters of cells in various states, expressing genes involved in various signaling pathways.

(C) Changes in proportion of each cell cluster over time for KAI-3 cell line after BCV treatment.

(D-F) Corresponding data shown for NK-S1 cell line.


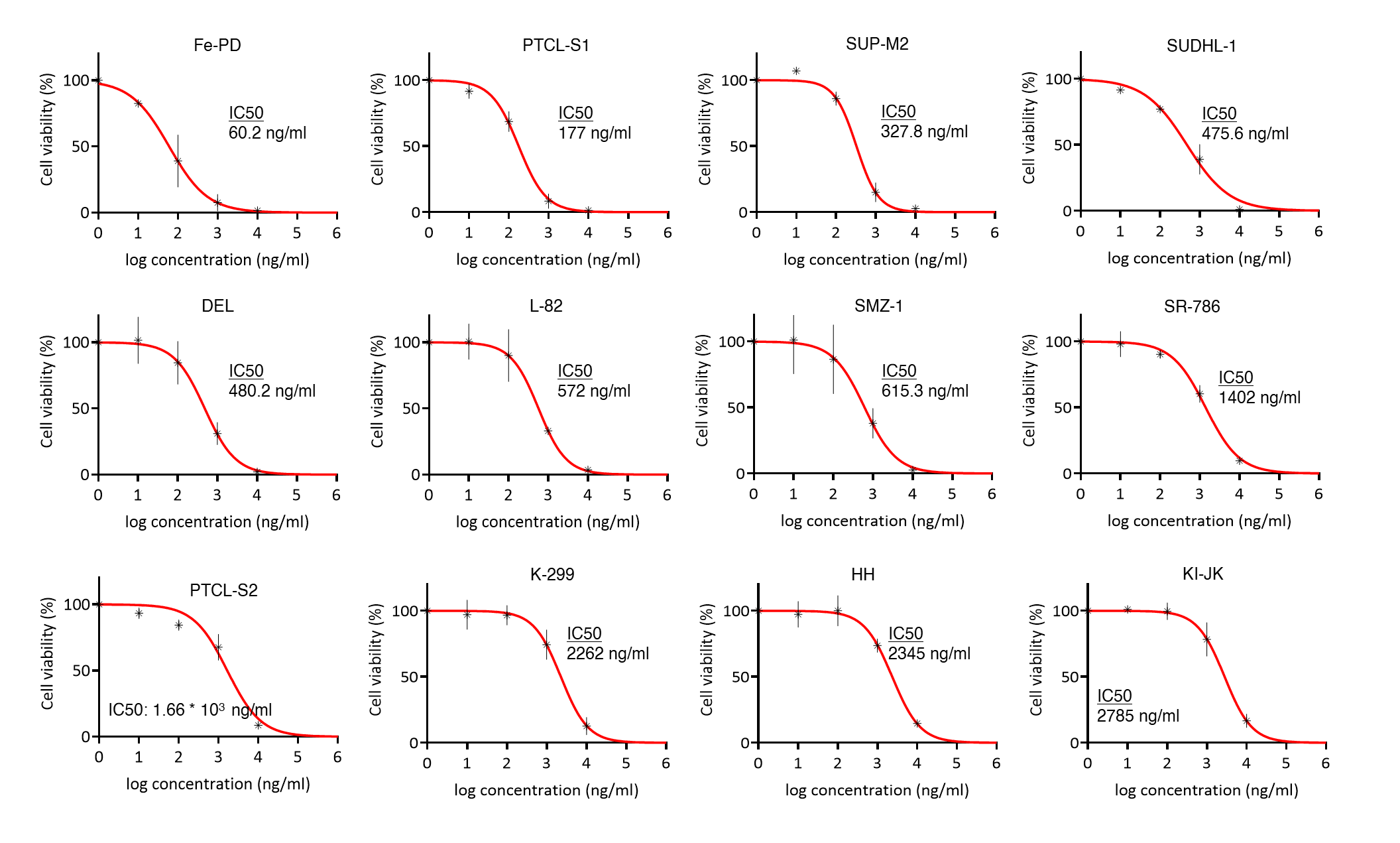


**Figure S5. Effect of BCV in T-cell lymphoma cell lines**


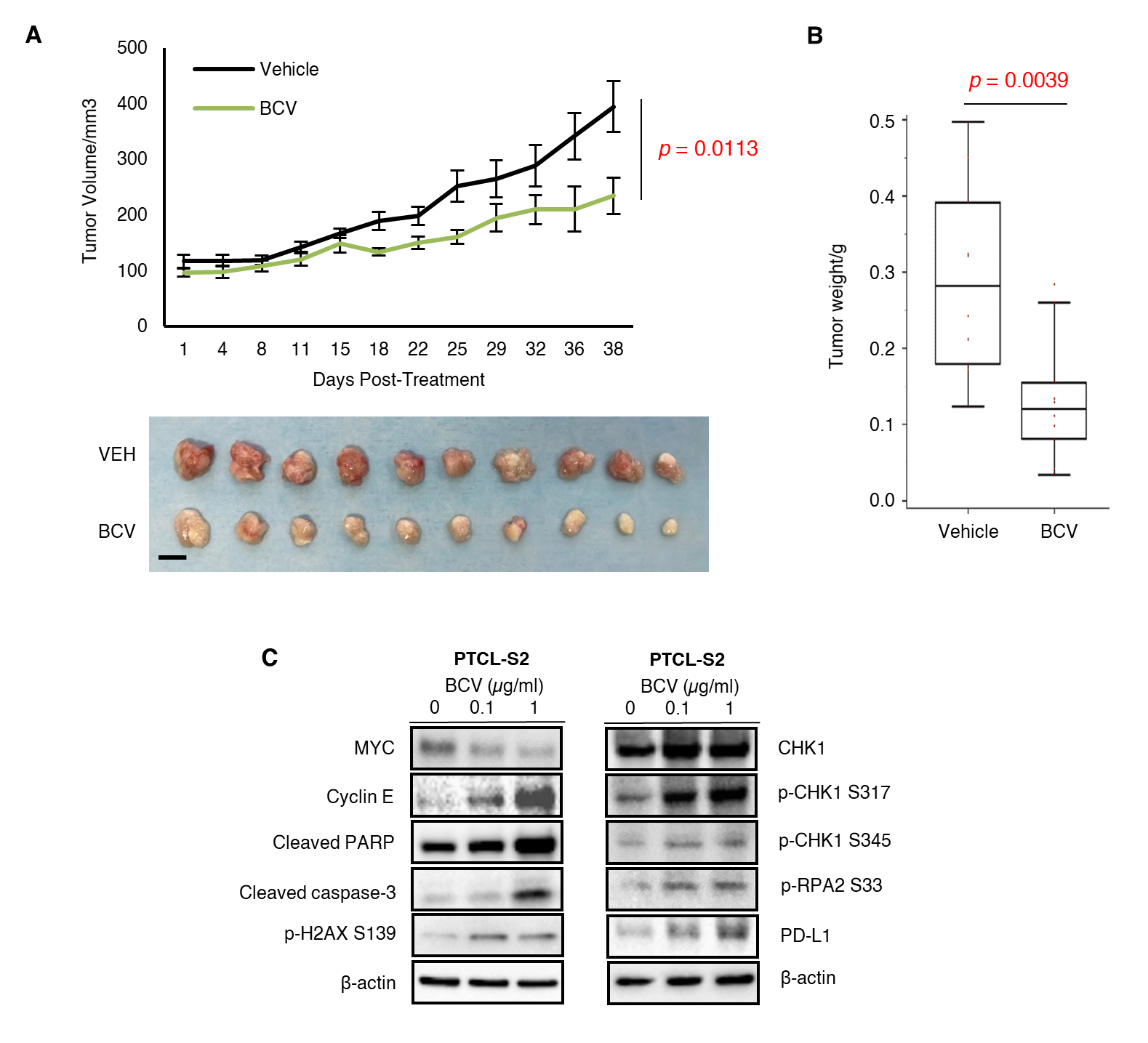


**Figure S6. In vivo efficacy of BCV in PTCL-S2 xenograft model**

(A) BCV inhibited tumor growth in the PTCL-S2 xenograft model (tumor volume: *p* = 0.0113, two-tailed t-test; (B) tumor weight: *p* = 0.0039, Mann-Whitney U test). NSG mice were treated twice per week IP with either vehicle or BCV (40 mg/kg) after subcutaneous flank inoculation with PTCL-S2 cells (n = 10 per group) (scale bar: 10 mm).

(C) Western blot demonstrated decrease protein expression of MYC, while cyclin E, cleaved PARP, cleaved caspase-3 were increased. p-H2AX, p-CHK1, p-RPA2, and PD-L1 were similarly increased.


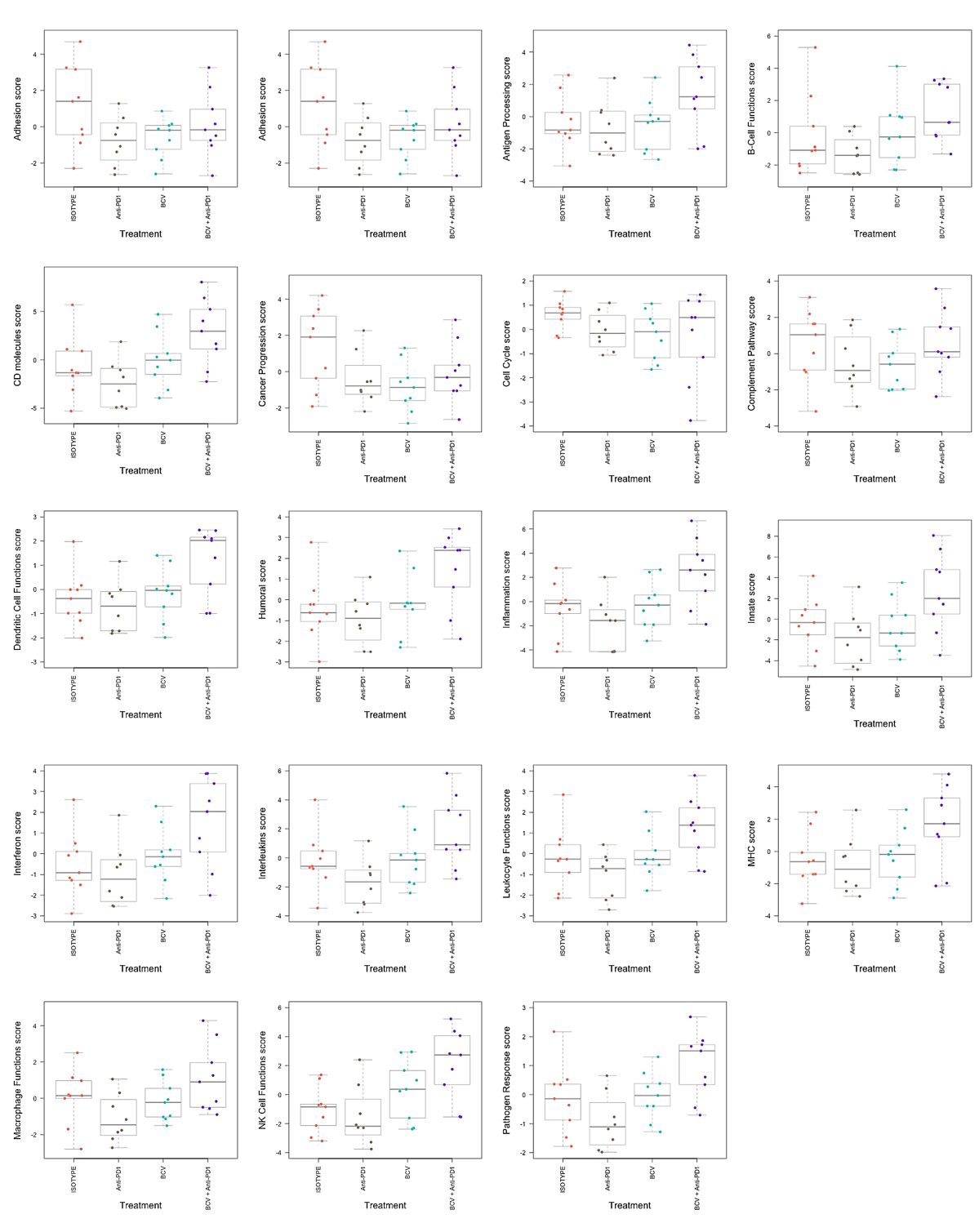


**Figure S7. NanoString pathway scores in EL4-C57BL/6 treatment groups**

**
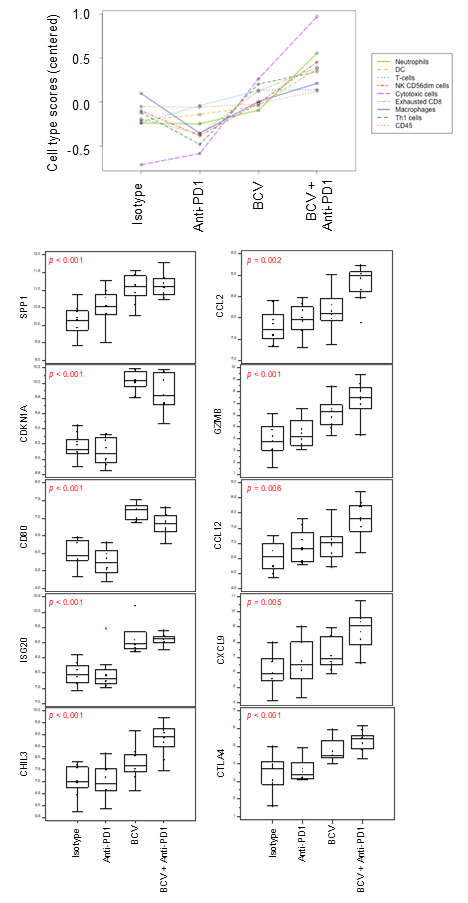
**

**Figure S8. NanoString cell-type scores and expression of selected genes in EL4-C57BL/6 treatment groups**


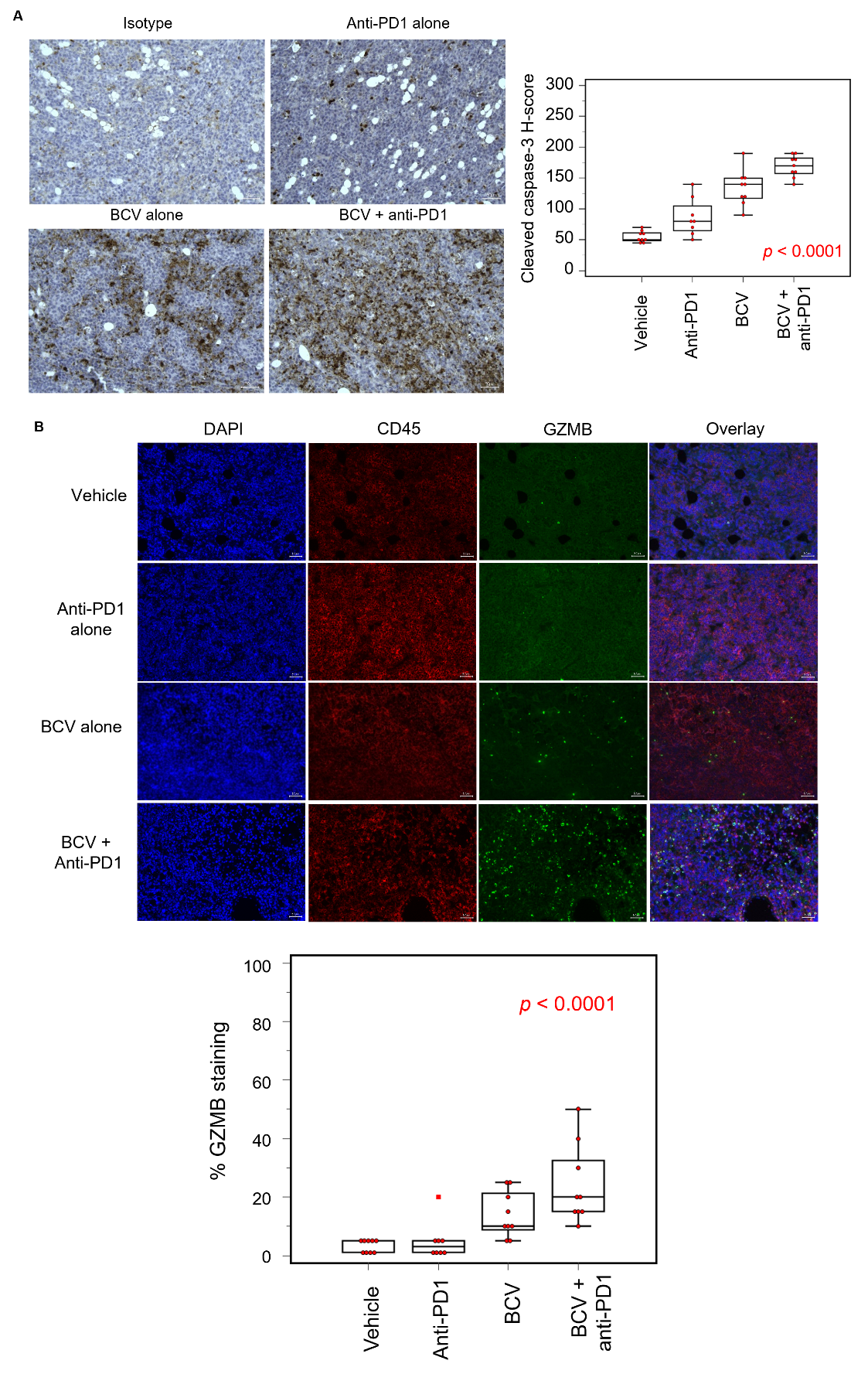


**Figure S9. Selected validation data for NanoString profiling results of EL4-C57BL/6 treatment groups**

(A) Representative images of cleaved caspase-3 IHC staining on EL4-C57BL/6 tumor specimens, showing increased apoptosis in BCV-treated tumors, especially in combination with anti-PD1 (scale bar: 50 µm).

(B) Representative images of GZMB immunofluorescence staining on EL4-C57BL/6 tumor specimens, showing significant GZMB-positive cells in tumors treated with BCV and especially in combination with anti-PD1 (scale bar: 50 µm).


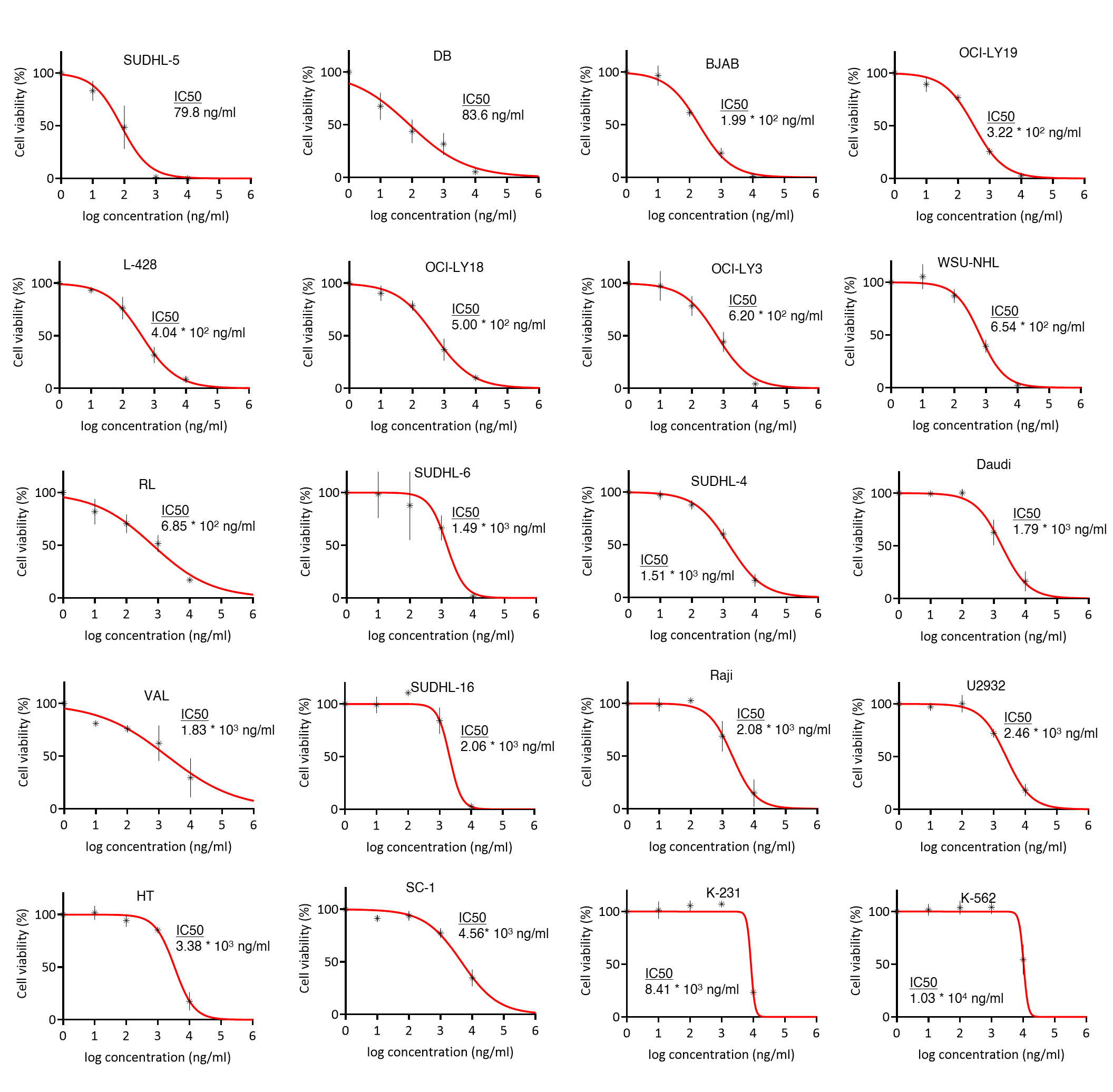


**Figure S10. Effect of BCV in B-cell lymphoma cell lines**


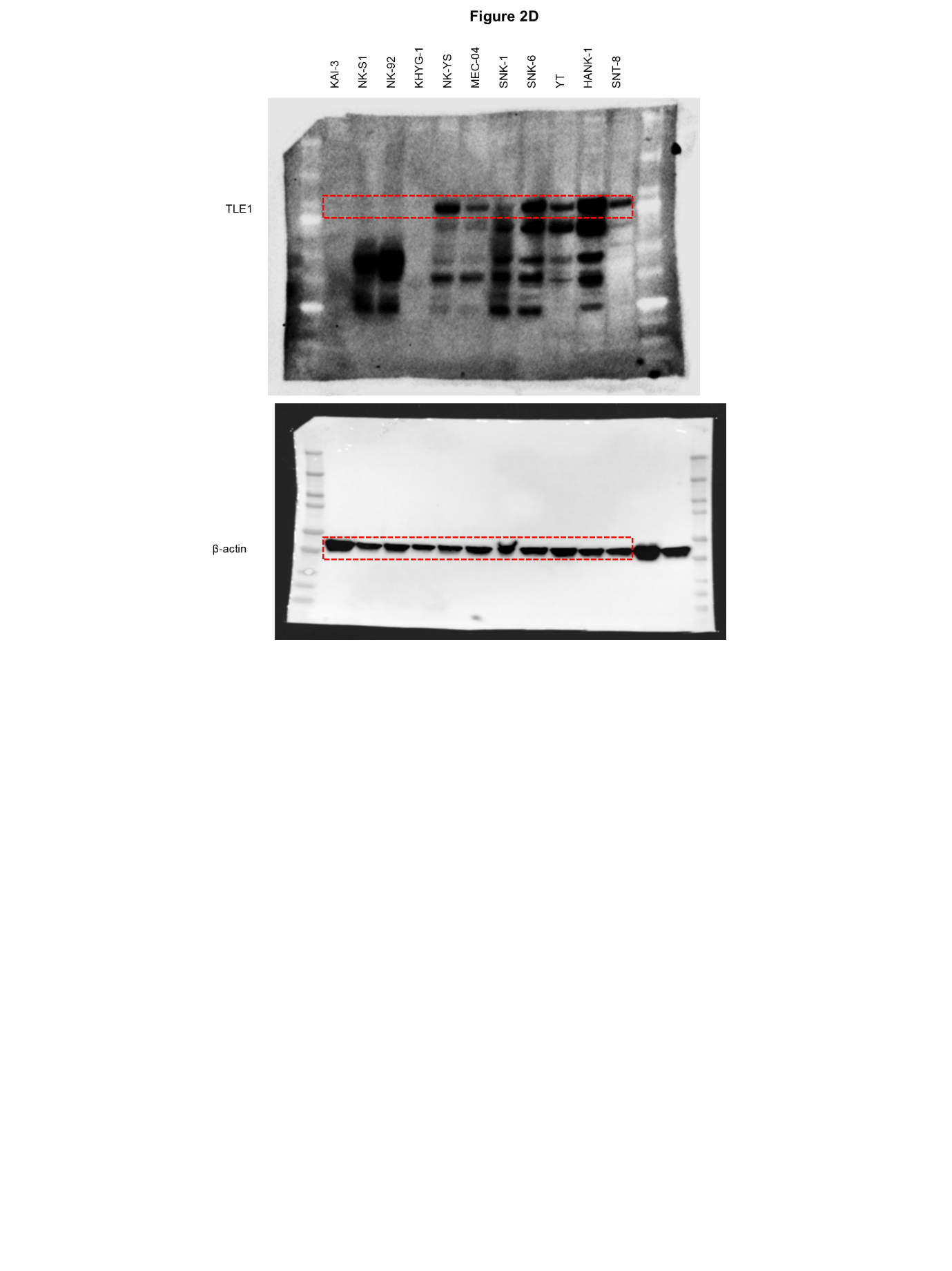


**Figure S11. Full gels for all Western blot images used in the manuscript**


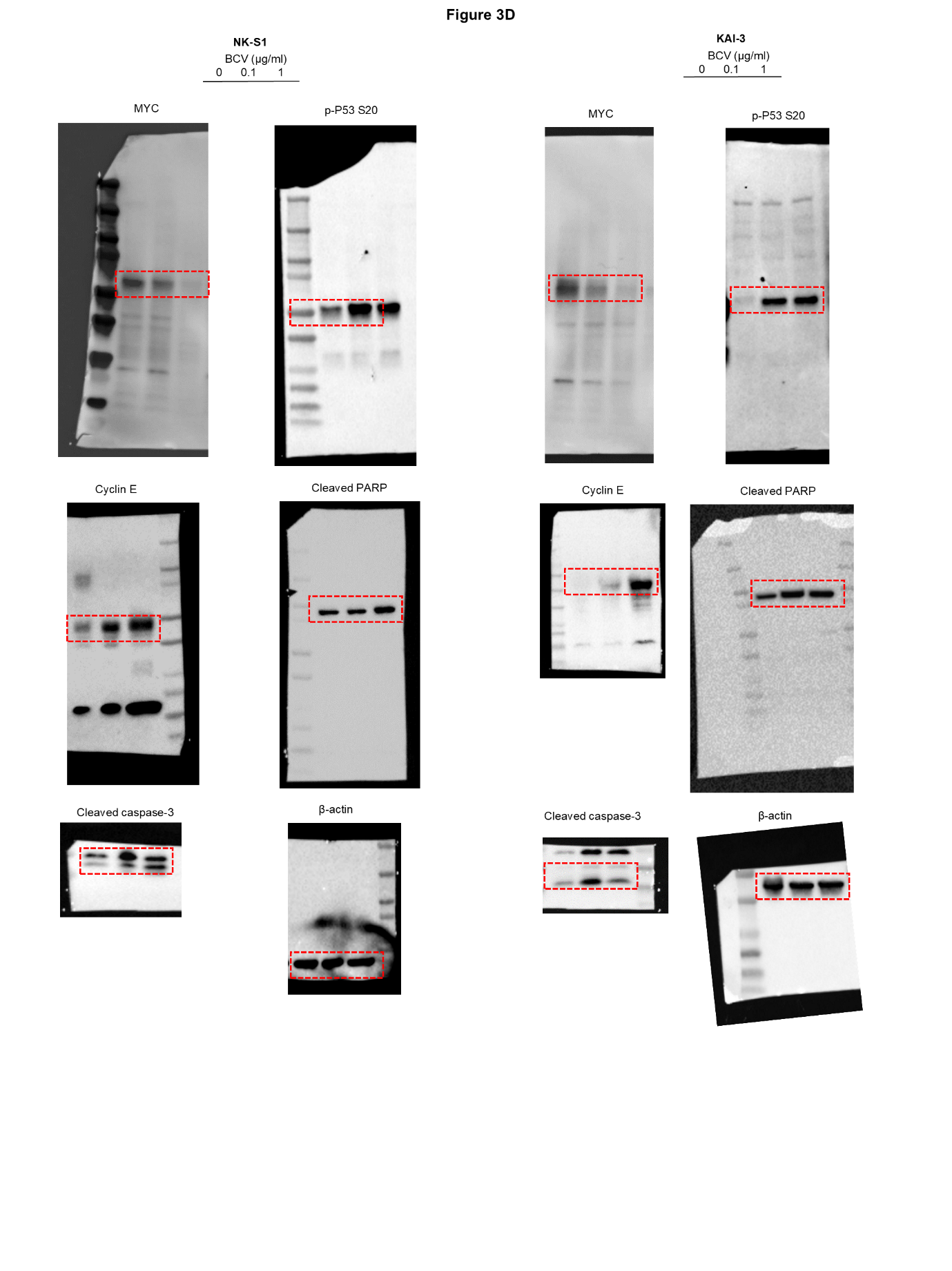


**Figure S11. Full gels for all Western blot images used in the manuscript (continued)**


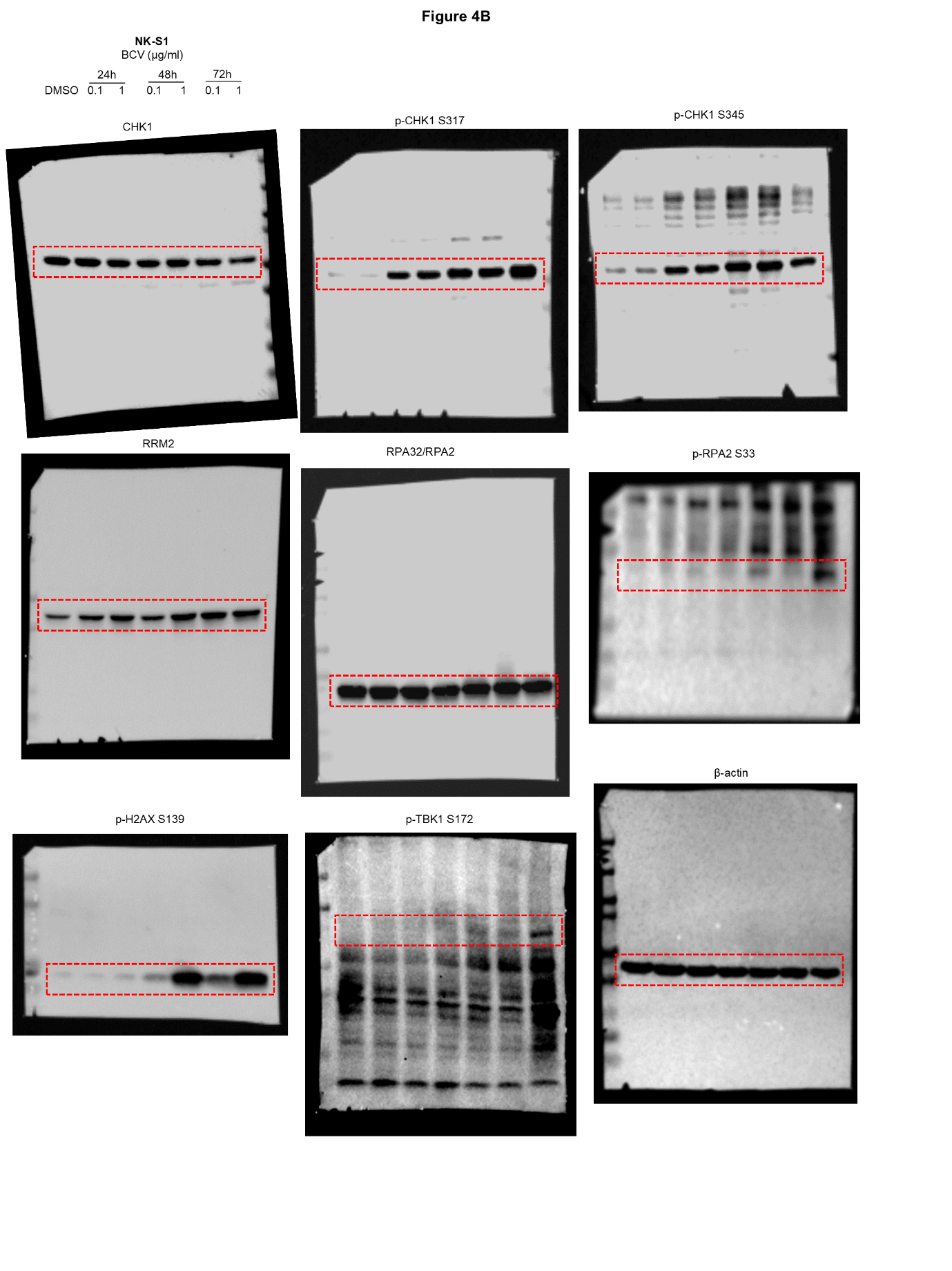


**Figure S11. Full gels for all Western blot images used in the manuscript (continued)**


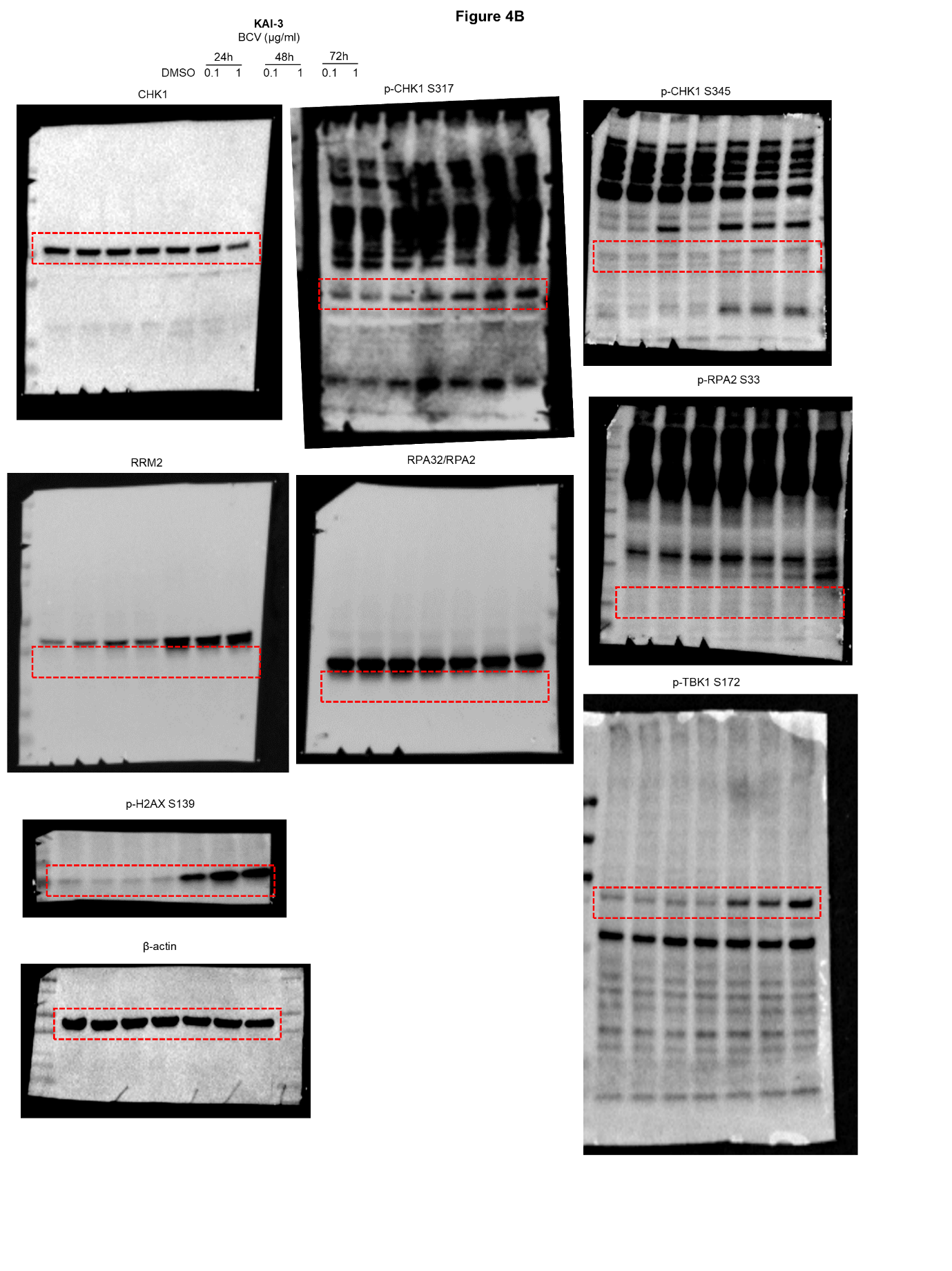


**Figure S11. Full gels for all Western blot images used in the manuscript (continued)**


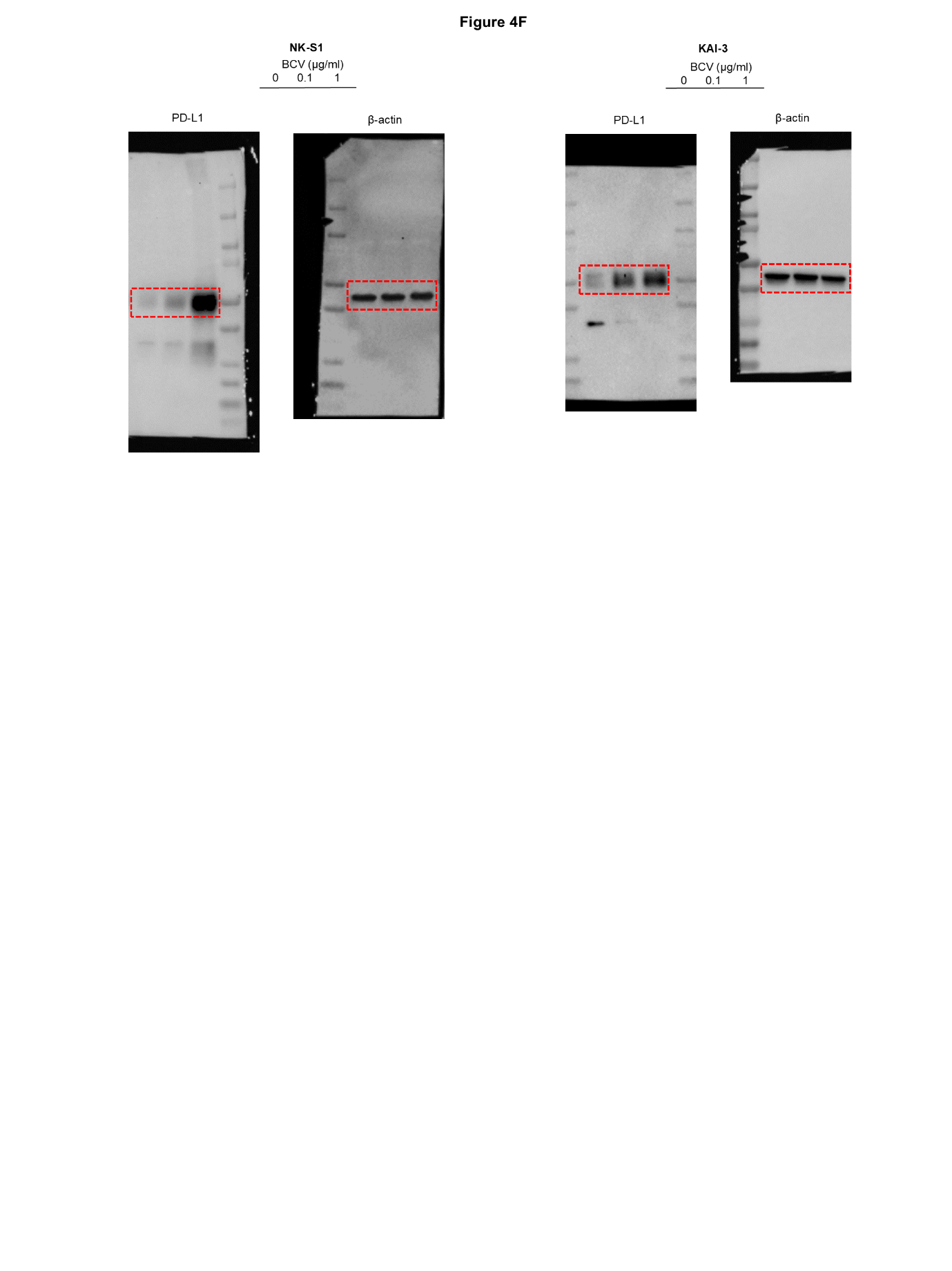


**Figure S11. Full gels for all Western blot images used in the manuscript (continued)**


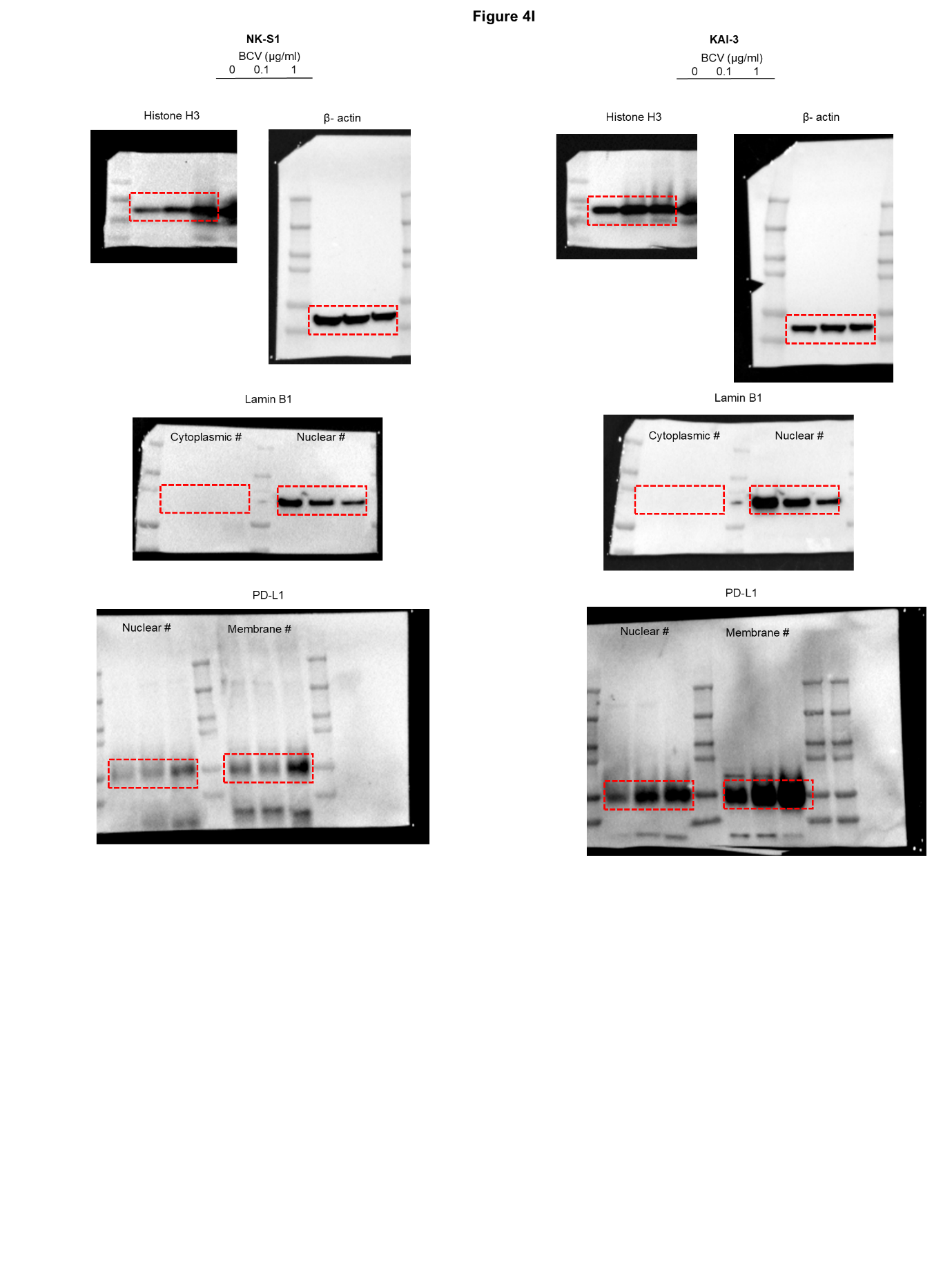


**Figure S11. Full gels for all Western blot images used in the manuscript (continued)**

**
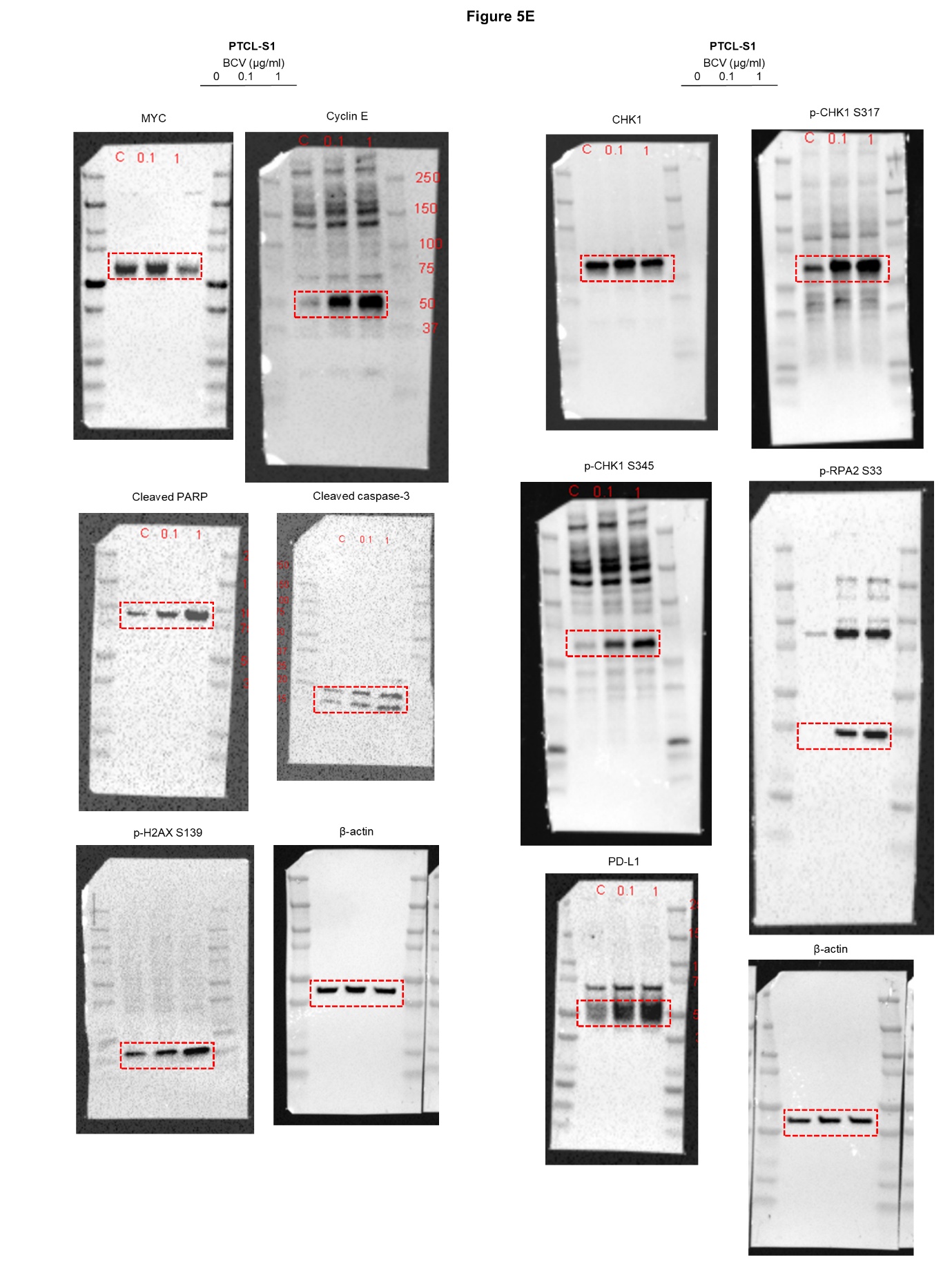
**

**Figure S11. Full gels for all Western blot images used in the manuscript (continued)**


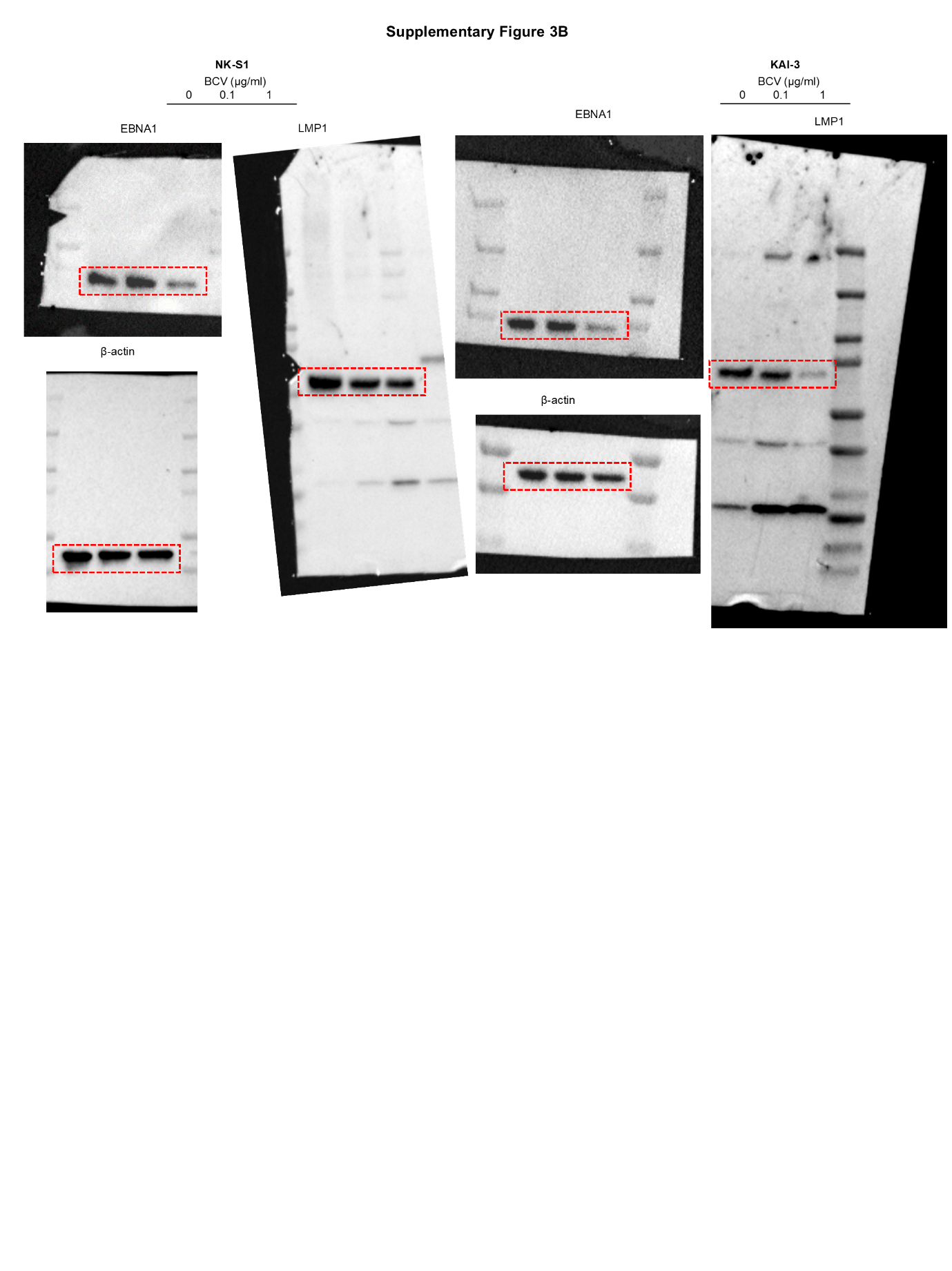


**Figure S11. Full gels for all Western blot images used in the manuscript (continued)**


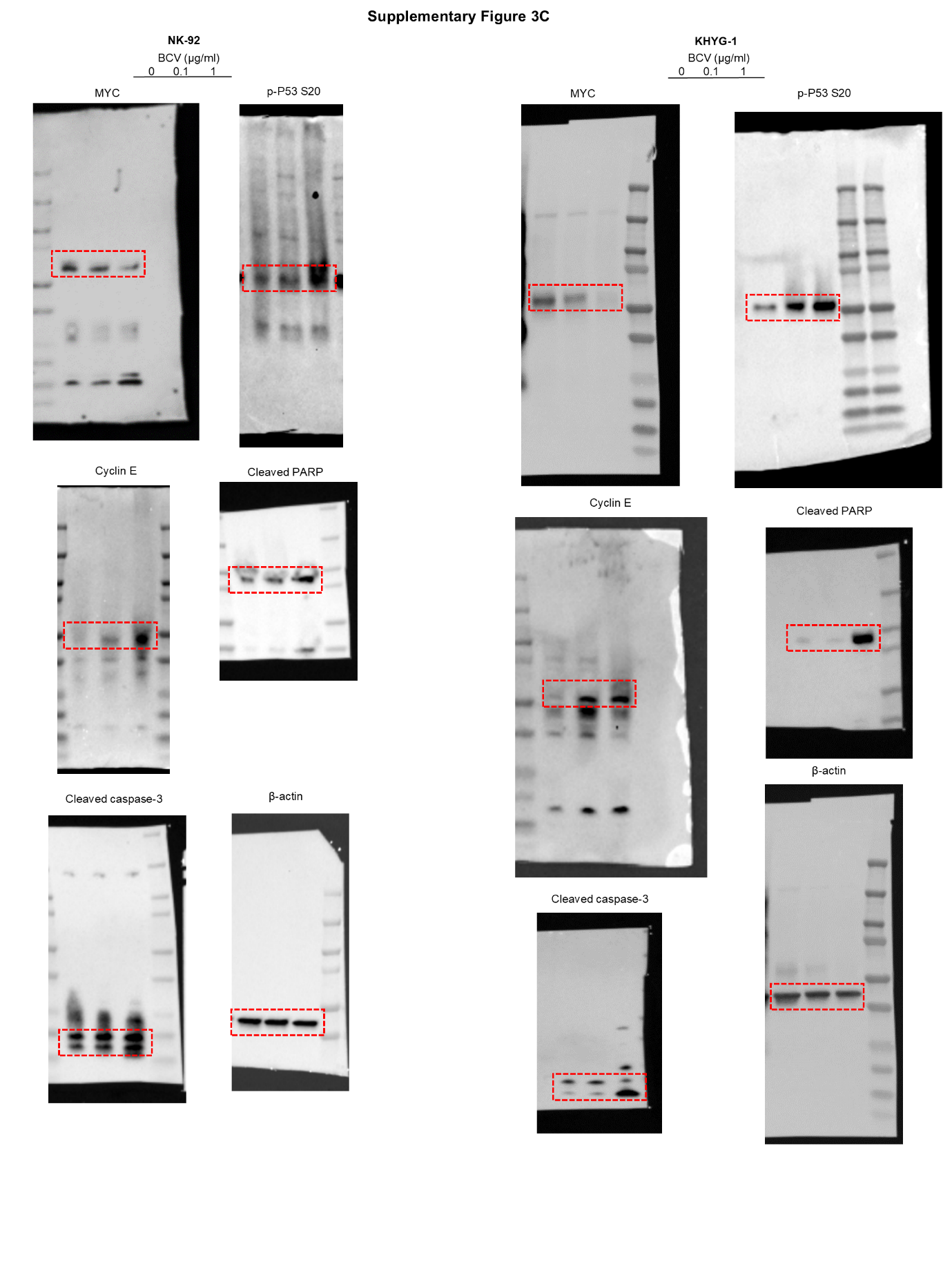


**Figure S11. Full gels for all Western blot images used in the manuscript (continued)**


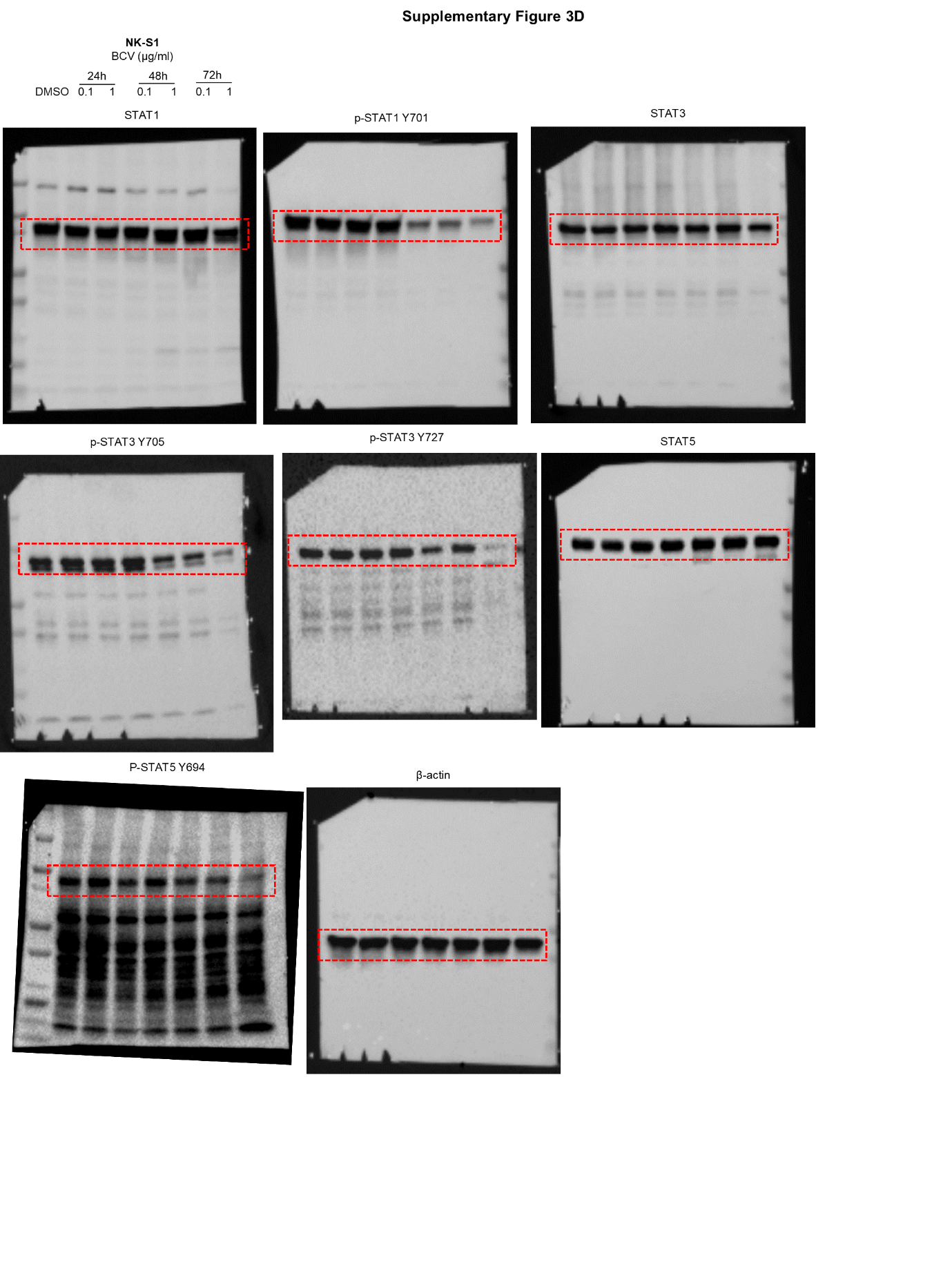


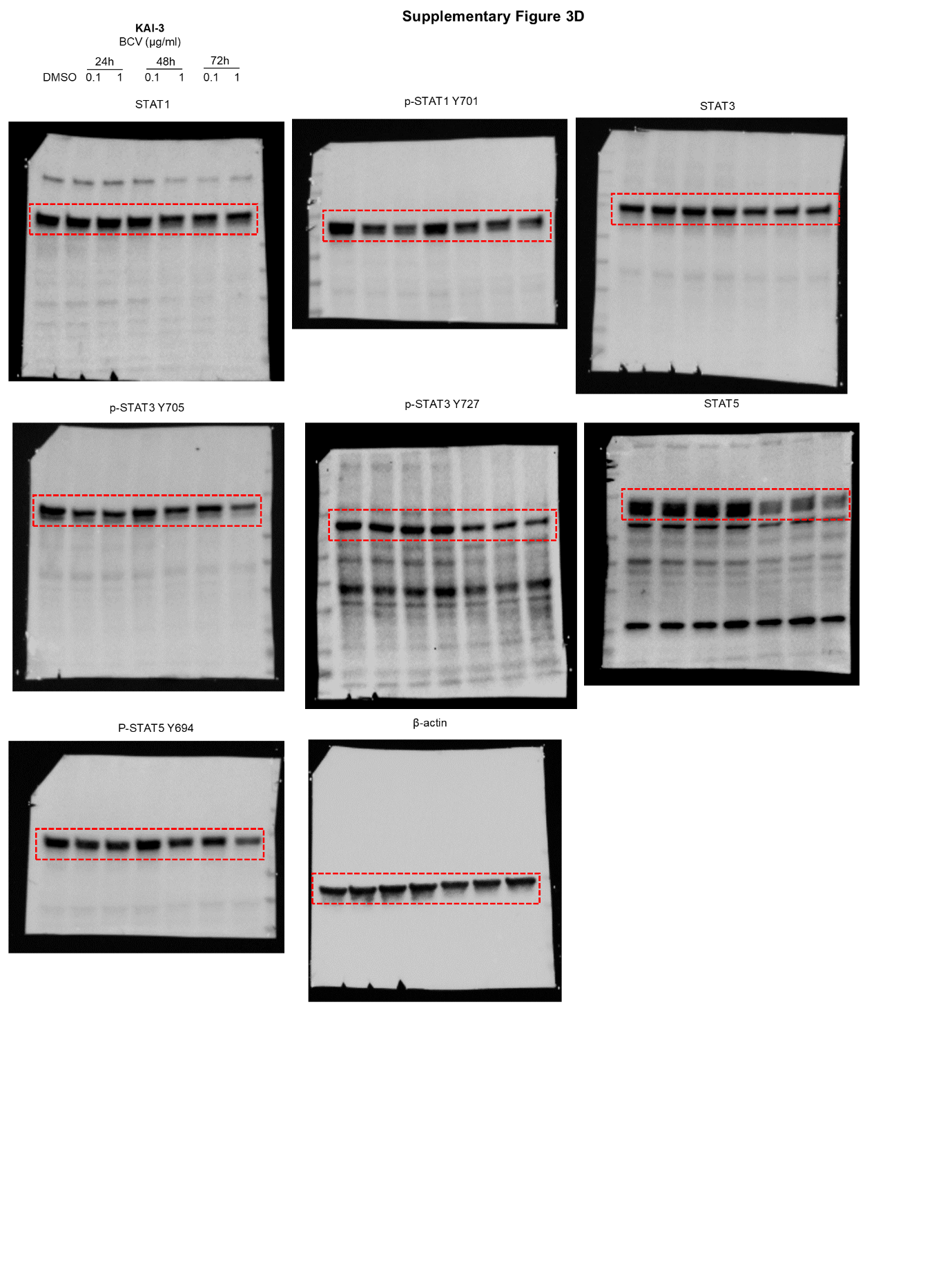


**Figure S11. Full gels for all Western blot images used in the manuscript (continued)**


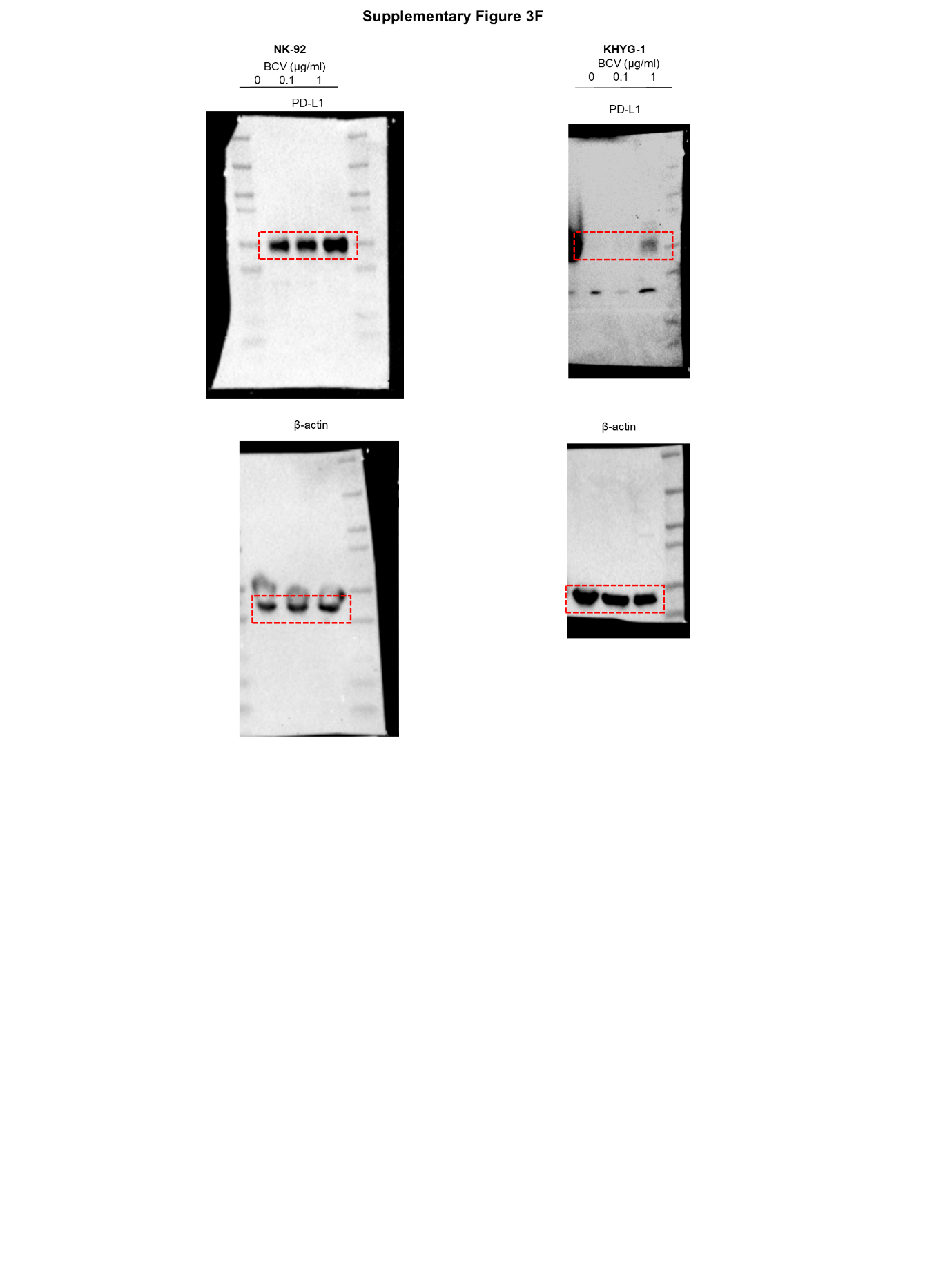


**Figure S11. Full gels for all Western blot images used in the manuscript (continued)**


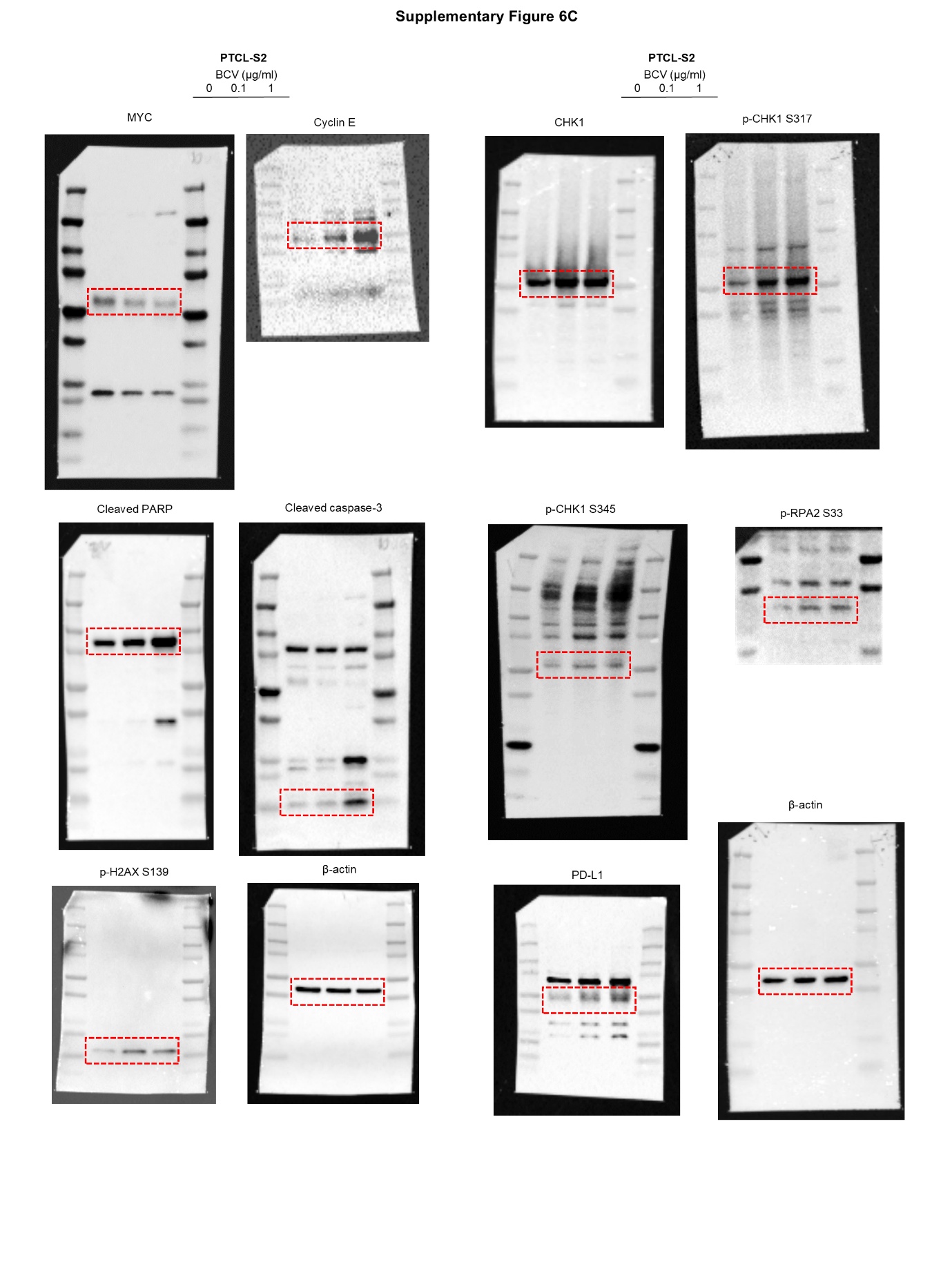


**Figure S11. Full gels for all Western blot images used in the manuscript (continued)**


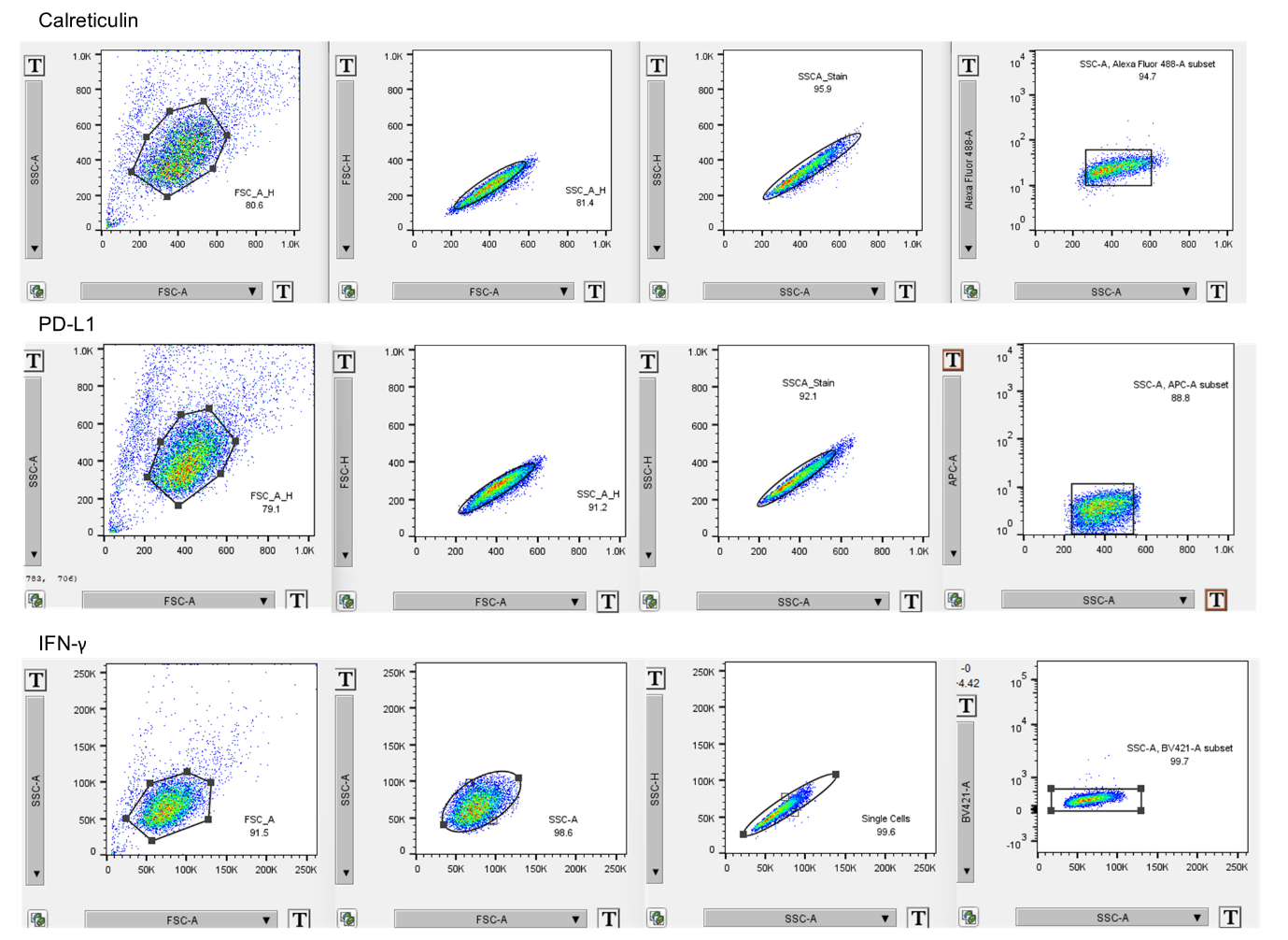


**Figure S12. FACS gating strategies.** Shown are representative FACS plots for calreticulin in figure 5D for NKS1 as an example, as well as for IFN-γ in figure S3E and PD-L1 in figure 4H.

**SUPPLEMENTARY METHODS**

***SDS-PAGE and Western blot***

Whole cell or cellular fraction lysates were obtained using Cell Fractionation Kit (#9038, Cell Signalling Technology, Danvers, MA, USA) following the manufacturers’ protocols. Cell lysates were subjected to separation using SDS-PAGE with 4 - 15% Mini-PROTEAN™ TGX Stain-Free™ Protein Gels (Bio-Rad Laboratories, Hercules, CA, USA) and then transferred onto 0.2 μm PVDF membranes (Bio-Rad Laboratories, Hercules, CA, USA). After blocking for 1 h with either 5% non-fat dry milk (Bio-Rad Laboratories, Hercules, CA, USA) or 5% bovine serum albumin (Sigma-Aldrich, Darmstadt, Germany) in TBST solution (50 mM Tris/HCl pH 7.4, 150 mM NaCl, 0.1% Tween-20), the membranes were exposed to primary antibodies (Additional file 2: Table S14) overnight at 4°C with gentle shaking. Exposure to the appropriate HRP-conjugated anti-rabbit or anti-mouse antibodies (Cytiva, Washington, DC, USA) were performed for 1 h and eventually subjected to chemiluminescence detection using the SuperSignal Substrate Western Blotting Kit (Thermo Fisher Scientific, MA, USA) and imaged with ChemiDoc™ XRS+ System with Image Lab™ Software (Bio-Rad Laboratories, Hercules, CA, USA). Expanded Western blot images used in the manuscript are shown in Additional file 1: Fig. S11.

***Quantification of gene expression via quantitative polymerase chain reaction (qPCR)***

Total RNA isolated as described above were subjected to cDNA synthesis using the iScript cDNA Synthesis kit, (Bio-Rad Laboratories, USA), according to the manufacturer’s protocol. Primer sequences are shown in Additional file 2: Table S14. qPCR was performed using Maxima SYBR Green/ROX qPCR Master Mix (Thermo Scientific, USA). ΔCt for mRNA expression of genes was calculated in relation to Ct value of GAPDH, which was used as an internal control. ΔΔCt was calculated by subtracting treated group ΔCt values from that of untreated control group. The normalised expression ratio was calculated with the formula 2^-ΔΔCt^.

***Immunohistochemistry***

Sections (4 μm) were cut from FFPE tissue blocks and mounted onto positively charged Bond Plus Slides (Leica Biosystems, Inc., Richmond, IL, USA) glass slides, and dried on a heating bench for at least 20 minutes. After deparaffinization and rehydration, tissue samples were subjected to antigen retrieval before incubation with antibodies (Additional file 2: Table S14). Slides were counter-stained with Mayer’s Haematoxylin (Dako, Glostrup, Denmark). Intensity of staining was quantified based on H-scores. A H-score is a semi-quantitative immunohistochemistry scoring method that incorporates both the intensity of staining and the percentage of cells stained at each intensity. For PD-L1, staining intensity was graded as absent (0), weak (1+), moderate (2+), or strong (3+). For each intensity category (0–3), the percentage of total cells exhibiting that staining was estimated, and the percentages for each sample must sum to 100%. For TLE1 staining, samples were arbitrarily deemed as TLE1 low on IHC with absent TLE1 staining, and TLE1 high on IHC with at least moderate staining pattern in >5% of the tumor cells.

***Confocal microscopy and identification of micronuclei***

Micronuclei identification was performed as previously described.^14^ Briefly, cells were fixed using 4% paraformaldehyde in PBS for 15 minutes, permeabilized with 0.1% Triton X-100 in PBS for 15 minutes and stained with NucBlue® Live ReadyProbes® Reagent (Hoechst 33342) (Thermo Fisher Scientific, USA) for 20 minutes and imaged with Leica TCS SP8 STED 3X (Leica Biosystems, Richmond, IL, USA). Confocal micrographs at 40X magnification of Hoechst 33342-stained cells were assessed for the occurrence of micronuclei by manual inspection. The criteria used for identifying micronuclei are: 1) round in shape; 2) less than a third of the size of a nucleus; 3) boundary of micronuclei is distinguishable from nuclear boundary; and 4) micronuclei are adjacent to a nucleus.

***Cell cycle analysis***

Fluorescence-activated cell sorting (FACS) analyses of sub-G_1_ fractions by using propidium iodide (PI) staining for DNA fragmentation. Briefly, the cells were harvested in 15-mL tubes, fixed with 70% ethanol, washed twice with PBS and stained with PI (50 μg/ml) and RNase A (100 μg/ml) for 30 minutes at 37°C. At least 10,000 events were analyzed by flow cytometry (Coulter EPICS Elite ESP; Beckman Coulter, Fullerton, CA, USA) with the excitation set at 488 nm and emission at 610 nm. Data were analyzed using Flowing Software version 2.5.1 (Turku Bioscience, Turku, Finland).

***Flow cytometry for IFN-γ, calreticulin and PD-L1 expression***

For PD-L1 and calreticulin staining, cells were incubated with respective antibody (1:100, 30 minutes at room temperature) (Additional file 2: Table S14), washed and resuspended in PBS. For PD-L1, this was marked with PE-conjugated secondary antibody (#31864, Thermo Fisher Scientific, MA, USA) before analysis (BD LSR Fortessa, BD Biosciences, San Jose, CA, USA). Calreticulin was conjugated to Alexa Fluor 488 (Abcam, Cambridge, UK). IFN-γ was conjugated with BV421 (#562988, BD Horizon, San Jose, CA, USA) and incubated at 1:200, 30 minutes at room temperature. Data were analyzed using FlowJo version 10.8.0 (BD Biosciences, San Jose, CA, USA). FACS gating strategies are available in Additional file 1: Fig. S12.

***HMGB1 release assay***

The release of High Mobility Group Box 1 (HMGB1) protein from cells was quantified using the Lumit HMGB1 (Human/Mouse) Immunoassay (Promega, Madison, WI, USA), as per manufacturer’s protocol. Briefly, 20 µl of 5X antibody mixture was added to 80 µl of treated cells and incubated for 60 minutes at room temperature. Following incubation, 25 µl of Lumit Detection Reagent B was added and luminescence recorded using Tecan M200 Infinite 96-well plate reader with IControl Software 1.6 (Tecan, Männedorf, Switzerland).

***NanoString gene expression profiling***

In order to correlate the observed phenotypic differences with transcriptomic alterations, gene expression profiling on murine EL4 cell line-derived tumors (from flank implants on C57BL6N mice) treated with BCV (n = 9), isotype alone (n = 9), anti-PD-1 (n = 8) and BCV plus anti-PD1 (n = 9), was performed using the NanoString Mouse Immunology Panel on the nCounter platform (NanoString Technologies, Seattle, WA, USA) following manufacturer’s protocol. RNA was extracted from all samples at the time of tumor harvest and analyzed using the 2100 Bioanalyzer (Agilent Technologies, Palo Alto, CA, USA). The data was analyzed on the nSolver 4.0 Advanced Analysis module using default settings to derive differentially-expressed genes, pathway scores, and cell-type scores.
